# The time interval from amyloid to tau PET positivity varies by age, sex and *APOE*-ε4 status

**DOI:** 10.1016/j.tjpad.2026.100622

**Published:** 2026-06-17

**Authors:** Marta Milà-Alomà, Isabella Hausle, Kellen K. Petersen, Pamela Thropp, Suzanne E. Schindler, Duygu Tosun

**Affiliations:** a Northern California Institute for Research and Education, San Francisco, CA, USA; b Department of Radiology and Biomedical Imaging, University of California San Francisco, San Francisco, CA, USA; c Department of Neurology, Washington University in St. Louis, St. Louis, MO, USA

**Keywords:** Alzheimer’s disease, Amyloid PET positivity, Tau PET positivity, Biological clock, Clinical trials, Symptom onset

## Abstract

**Background:**

Alzheimer’s disease (AD) progression varies widely among individuals. Identifying factors influencing timing of pathology and clinical progression is crucial for optimizing early intervention trials.

**Objectives:**

To investigate how the estimated age at amyloid and tau PET positivity, and the time interval between these two key events (“amyloid–tau time interval”), relate to symptom onset and clinical progression, and to assess the effects of *APOE*-ε4 status and sex on these associations.

**Design:**

This analysis used data from the Alzheimer’s Disease Neuroimaging Initiative (ADNI) and the Harvard Aging Brain Study (HABS).

**Setting:**

The ADNI is a multicenter observational cohort conducted at 55 sites across the United States; The HABS is a longitudinal, single-center observational cohort.

**Participants:**

This study included participants with at least one positive amyloid PET scan (ADNI *n* = 792; HABS *n* = 104) or at least one positive tau PET scan (ADNI *n* = 212; HABS *n* = 48). All participants had information on sex, *APOE-*ε4 status, and longitudinal cognitive assessments.

**Measurements:**

We examined the influence of *APOE*-ε4 status, sex, and their interaction on the estimated age at biomarker positivity and the amyloid-tau time interval. Accelerated Failure Time (AFT) models were used to predict time to symptom onset (CDR > 0) based on estimated biomarker positivity age and the amyloid-tau time interval. Linear mixed-effects (LME) models evaluated differences in the rate of cognitive decline, as measured by CDR-SB, over five years following symptom onset according to estimated biomarker positivity age and amyloid-tau time interval. Additional models included interaction terms with sex or *APOE*-ε4 status.

**Results:**

The amyloid-tau time interval varied markedly between individuals and was shorter in *APOE-*ε4 carriers, women, and those with older age at amyloid PET positivity. *APOE-*ε4 carriers and women became amyloid and tau PET positive at younger ages. Following amyloid PET positivity, a shorter time to tau PET positivity predicted earlier symptom onset. After symptom onset, faster cognitive decline was observed in individuals with younger ages at amyloid or tau PET positivity. The time to symptom onset following tau PET positivity, or the rate of cognitive decline after symptom onset, were not influenced by the amyloid-tau time interval.

**Conclusions:**

After becoming amyloid PET positive, *APOE-*ε4 carriers, women and older individuals may have a shorter window for detection and treatment before they become tau PET positive and develop symptoms. These findings should guide the identification of individuals at highest risk of rapid AD progression, enabling more efficient participant selection for clinical trials.

## 1 Introduction

Alzheimer’s disease (AD) is characterized by the accumulation of amyloid-β (Aβ) plaques and tau neurofibrillary tangles, which begin years before development of clinical symptoms [1,2]. Amyloid deposition typically precedes widespread tau aggregation [1], and both pathologies accumulate relatively consistently across individuals [1,3,4]; however, interindividual variability exists in the onset and temporal sequence of amyloid and tau pathologies and their contribution to clinical progression [3,4]. Understanding the factors driving this variability is essential for improving disease staging, prognosis, and treatment timing.

Positron emission tomography (PET) imaging enables the *in vivo* quantification of amyloid and tau pathologies. PET-based modeling can estimate an individual’s age at which a specific biomarker positivity threshold is crossed [3,4]. These estimated ages for PET positivity offer a valuable tool for studying the temporal dynamics of AD pathophysiology at an individual level and identifying factors influencing key pathological milestones. Anchoring analyses to an individual’s biological disease timeline may also enhance the prediction of clinical outcomes, such as symptom onset or rate of cognitive decline.

Although advanced age is the primary risk factor for AD, genetic and demographic factors such as the apolipoprotein E (*APOE*) genotype and sex significantly contribute to disease risk and progression [5]. The *APOE-*ε4 allele, the strongest genetic risk factor for late-onset AD, is consistently associated with earlier amyloid and tau accumulation, and earlier development of clinical symptoms [6]. Sex differences have also been reported in AD prevalence and progression, with men showing less vulnerability to tau pathology and slower clinical decline than women [[7], [8], [9], [10], [11], [12]]. Furthermore, interactions between *APOE*-ε4 and sex have been observed to influence AD risk and progression [[13], [14], [15]].

Despite these established connections, it remains unclear to what extent *APOE-*ε4 status and sex, independently and interactively, influence the timing of amyloid and tau PET positivity, and how these temporal dynamics influence the disease course. While amyloid positivity is necessary, the emergence of tau and subsequent cognitive decline signal imminent clinical progression [[16], [17], [18], [19], [20]]. A better understanding of factors influencing the time interval from reaching amyloid positivity to subsequent tau positivity, which we term “the amyloid-tau time interval”, could identify individuals at higher risk of rapid progression once amyloid positivity is established. This has significant implications for early-stage clinical trials, which often enroll participants based solely on amyloid burden. Refining participant selection by considering both amyloid burden and individual trajectories toward tau positivity and symptom onset could improve trial efficiency and therapeutic impact. Investigating how *APOE-*ε4 status and sex impact these key events can offer valuable insight into the mechanisms driving variability in tau emergence, with direct implications for prognostic modeling and selection of individuals for early intervention in clinical trials.

This study investigated how the estimated age at amyloid and tau PET positivity, and the amyloid-tau time interval, relate to symptom onset and clinical progression. We also assessed the independent and interactive effects of *APOE-*ε4 status and sex on these associations. By characterizing how these factors modulate the temporal sequence of events in AD pathogenesis, we aim to better understand individual variability in AD progression and its potential clinical consequences.

## 2 Methods

### 2.1 Study participants

This study included cognitively unimpaired (CU) and cognitively impaired (CI) participants from the Alzheimer’s Disease Neuroimaging Initiative (ADNI) study (adni.loni.usc.edu), which served as the primary cohort for all analyses. ADNI is a public-private partnership focused on identifying biomarkers for mild cognitive impairment (MCI) and early AD progression [21,22]. The Harvard Aging Brain Study (HABS) [23] was included as an independent replication cohort to evaluate the generalizability of findings observed in ADNI. The overall goal of the HABS is to elucidate the earliest changes in molecular, functional and structural imaging markers that signal the transition from normal cognition to progressive cognitive decline along the trajectory of preclinical AD.

For this study, we required at least one positive amyloid PET scan or tau PET scan to estimate biomarker positivity ages (Supplementary Methods). All included participants had longitudinal cognitive assessments, self-identified sex, and *APOE* genotype data.

### 2.2 Standard protocol approvals, registrations, and patient consents

All participants gave written informed consent to participate in the ADNI or the HABS as approved by the ethics committees of all participating sites. All methods were carried out in accordance with the approved guidelines.

### 2.3 Amyloid and tau PET measures

In ADNI, amyloid PET imaging used ^18^F-florbetapir (FBP) or ^18^F-florbetaben (FBB) with standardized ADNI protocols [24]. Global cortical standardized uptake value ratio (SUVR) was calculated by averaging PET uptake across cortical regions (frontal, cingulate, parietal, and lateral temporal) and normalizing to a composite reference region (whole cerebellum, brainstem/pons, and subcortical eroded white matter) [25]. Amyloid PET positivity was defined as SUVR > 0.78 for FBP or SUVR > 0.74 for FBB, per ADNI guidelines (ADNI_UCBerkeley_AmyloidPET_Methods_v2_2023-06-29.pdf). Tau PET imaging used ^18^F-flortaucipir (FTP) with standardized ADNI protocols. Mesial-temporal meta-region of interest (ROI) uptake (including entorhinal, parahippocampus, and amygdala) was normalized to the inferior cerebellar grey matter uptake [26]. We determined mesial-temporal tau PET positivity threshold of 1.41 SUVR using a two-component Gaussian mixture model (GMM) applied to cross-sectional mesial-temporal FTP PET SUVR from the entire ADNI study (*n* = 907).

In HABS, ^11^C-Pittsburgh compound B (PiB) PET acquisition parameters have been previously published [23,27]. Distribution volume ratios (DVRs) were calculated via Logan plotting over 40 to 60 minutes with a cerebellar gray matter reference region and a global aggregate was calculated by averaging frontal, lateral temporal, and retrosplenial (FLR) regions, as previously reported [14,28]. Amyloid PET positivity was defined as DVR > 1.2 [28]. FTP PET acquisition parameters for HABS have been reported previously [29]. A mesial-temporal meta-ROI was calculated using the same approach as described for ADNI. Mesial-temporal tau PET positivity was defined using a GMM-derived threshold of 1.27 SUVR.

### 2.4 Clinical and neuropsychological assessments

In both ADNI and HABS, participants underwent comprehensive clinical assessments, including collateral interviews, neurological examinations, and evaluation of global and domain-specific cognitive performance [23,30].

Participants were classified as CI if they had two consecutive visits with a Clinical Dementia Rating® (CDR) > 0 and a CDR > 0 in their last visit. Participants who reverted to a CDR = 0 were excluded. Symptom onset was defined as the first visit with a CDR > 0. For the purposes of this study, we used the CDR-Sum of Boxes (CDR-SB) [31] as the primary cognitive outcome due to its relevance as a clinical trial endpoint. The Digit Symbol Substitution Test version of the modified Preclinical Alzheimer Cognitive Composite (mPACC) [32] was additionally included for descriptive purposes.

### 2.5 Statistical analyses

We estimated ages at amyloid and tau PET positivity using a previously validated method for FBP PET and FTP PET in ADNI [3,33]. We extended this approach to ADNI FBB PET data (in 19.9% of participants with amyloid PET), showing similar performance (Supplementary Methods, Supplementary Fig. S1, S2). The same method was applied to HABS ^11^C-PiB and FTP PET data to estimate amyloid and tau PET positivity ages in this cohort (Supplementary Fig. S3, S4).

We used linear regression to assess the independent effects of *APOE-*ε4 status (*APOE-*ε4 carrier *vs. APOE-*ε4 non-carrier) and sex on estimated amyloid and tau PET positivity ages. Interaction terms between *APOE-*ε4 status and sex were evaluated in separate models.

The amyloid-tau time interval was calculated by subtracting the estimated age at amyloid PET positivity from the estimated age at tau PET positivity. We used linear regression to examine the effects of *APOE-*ε4 status, sex, and their interaction on the amyloid-tau time interval. Additional models included the estimated age at amyloid PET positivity and its interaction with *APOE-*ε4 status, sex, and the three-way interaction. To contrast radiological and biochemical disease intervals, we conducted a sensitivity analysis in ADNI integrating longitudinal plasma p-tau217 data (Fujirebio Lumipulse p-tau217). We modeled a plasma p-tau217 clock using a GMM-derived positivity cutoff of 0.257 pg/mL to estimate the age at plasma p-tau217 positivity [34] (Supplementary Methods, Supplementary Fig. S5). We then calculated the amyloid-plasma p-tau217 time interval and compared the duration of this biochemical interval to the radiological amyloid-tau PET time interval.

We used Accelerated Failure Time (AFT) survival models with a Weibull distribution to assess how estimated biomarker positivity ages and the amyloid-tau time interval influenced time to symptom onset (from CDR = 0 to CDR > 0) following amyloid or tau PET positivity. The time-to-event variable was the difference between age at CDR > 0 and estimated amyloid or tau PET positivity age. Individuals who did not convert to CDR > 0 during follow-up were included as right-censored observations at their last cognitive assessment. Sex, *APOE-*ε4 status, and educational attainment were included as covariates. Additional models tested interactions of estimated biomarker positivity ages or amyloid-tau time interval with sex, *APOE-*ε4 status, and their three-way interaction.

Lastly, we used linear mixed effect (LME) models with random slopes and intercepts to assess the effect of estimated biomarker positivity age or amyloid-tau time interval on the rates of cognitive decline, measured by CDR-SB, over five years following symptom onset (defined as the first visit with a CDR > 0). For these analyses, we grouped participants by estimated biomarker positivity age ( < 65, 65-75, and > 75 years) and by the amyloid-tau time interval. The latter was categorized as: “concurrent amyloid-tau” (−4 ≤ interval ≤ 4 years, reflecting the cumulative mean absolute error for estimated biomarker positivity ages; see Supplementary Methods), “amyloid first ≤ 10 years” (4 < interval ≤ 10 years), and “amyloid-first > 10 years” (interval > 10 years). Interaction terms between time since symptom onset and estimated biomarker positivity age group or amyloid-tau time interval group were evaluated. Next, pairwise contrasts were conducted to compare group-specific cognitive decline rates. We repeated these analyses adding interactions between time, estimated biomarker positivity age group or amyloid-tau time interval group and sex or *APOE*-ε4 status. Six participants with tau PET positivity preceding amyloid PET positivity (interval < −4 years; “tau-first” group) were excluded for the LME analyses comparing rates of cognitive decline across amyloid-tau time interval groups due to the very small sample size of this subgroup; however, these individuals were retained in all other analyses and included in the descriptive results.

We conducted sensitivity analyses in ADNI using baseline chronological age instead of estimated biomarker positivity age in linear regression analyses of the amyloid-tau time interval and in LME models evaluating differences in clinical progression across age groups. These analyses were performed to assess the specificity of biomarker-derived age measures beyond chronological age in capturing disease progression milestones.

Power analyses were conducted to evaluate whether the HABS sample size was sufficient to detect the effect sizes observed in ADNI. Required sample sizes were estimated based on ADNI-derived effect sizes and the HABS study design. Replication analyses in the HABS cohort were performed for those outcomes where adequate power was available.

All statistical analyses and figure generation were performed using R software (R version 4.2.2).

### 2.6 Data availability

Data from this study and the study methodology report may be accessed from the ADNI Laboratory of NeuroImaging (LONI) database: adni.loni.usc.edu. Access the HABS data may be requested at habs.mgh.harvard.edu/researchers/request-data/. The annotated code used for study analyses is provided in full (https://github.com/cind/Amyloid-tau-interval-paper-Mil-Alom).

## 3 Results

### 3.1 Study participants characteristics and estimated ages at amyloid and tau PET positivity

**Fig. 1:**
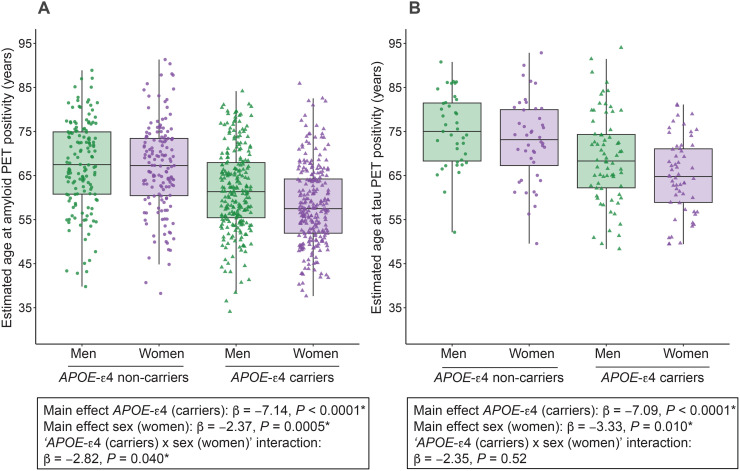
**Estimated ages at amyloid (A) and tau (B) PET positivity by *APOE*-ε4 status and sex**. Box plots depict the median (horizontal bar), interquartile range (IQR; box), and 1.5 × IQR (whiskers). Beta coefficients and *P*-values from linear regression models are shown for the main effects of *APOE*-ε4 status (carriers *vs*. non-carriers) and sex (women *vs*. men). Interaction terms between *APOE*-ε4 status and sex were assessed in separate models, and their corresponding beta coefficients and *P*-values are also reported.

There were 792 ADNI participants who had an estimated amyloid PET positivity age and 212 who had an estimated tau PET positivity age; 152 participants had both estimates. Demographic and clinical characteristics were similar between these subgroups (Table 1). The mean estimated amyloid and tau PET positivity ages were 63.3 ± 10.2 years and 69.9 ± 9.9 years, respectively. Both *APOE*-ε4 carriership and female sex were significantly associated with earlier estimated biomarker positivity ages (Fig. 1A, B). A significant *APOE*-ε4 status by sex interaction was found for amyloid PET positivity age (*P* = 0.040), with the association between *APOE*-ε4 carriership and earlier amyloid PET positivity age being stronger in women (Fig. 1A). No significant interaction effect was observed for tau PET positivity age (*P* = 0.52; Fig. 1B).

**Table 1:** Participant characteristics at first PET acquisition.

	**Cohort with estimated age at amyloid PET positivity (*****N*****= 792)**	**Cohort with estimated age at tau PET positivity (*****N*****= 212)**	**Cohort with PET positivity age for both amyloid and tau (*****N*****= 152)**
Age, years	74.3 ± 7.3	76.3 ± 7.8	73.9 ± 6.8
Women, n (%)	399 (50.4)	103 (48.6)	74 (48.7)
*APOE*-ε4 carriers, n (%)	493 (62.2)	127 (59.9)	104 (68.4)
Educational attainment (High school or less / College / Postgraduate), n (%)	120 (15.2) / 350 (44.2) / 322 (40.6)	20 (9.4) / 103 (48.6) / 89 (42.0)	17 (11.2) / 74 (48.7) / 61 (40.1)
Ethno-racial background (Black / White / Other), n (%)	39 (4.9) / 728 (91.9) / 25 (3.2)	8 (3.8) / 189 (89.1) / 15 (7.1)	6 (3.9) / 136 (89.5) / 10 (6.6)
CDR (0 / 0.5 / > 0.5), n (%)	248 (31.3) / 453 (57.2) / 91 (11.5)	72 (34.0) / 113 (53.3) / 27 (12.7)	54 (35.5) / 91 (59.9) / 7 (4.6)
CDR-SB	1.75 ± 2.11	1.83 ± 2.25	1.25 ± 1.49
mPACC	-6.53 ± 7.11	-7.09 ± 7.38	-5.62 ± 6.20
**Vascular risk factors**^a^			
Diabetes, n (%)	47 (9.4)	5 (7.4)	5 (10.9)
Hypertension, n (%)	335 (42.3)	92 (43.4)	70 (46.1)
Obesity, n (%)	146 (18.4)	36 (17.0)	32 (21.2)
Dyslipidemia, n (%)	179 (35.9)	26 (38.2)	15 (32.6)
Chronic kidney disease, n (%)	126 (25.3)	11 (16.2)	7 (15.2)
Total white matter hyperintensity volume, %	0.49 ± 0.70	0.49 ± 0.83	0.43 ± 0.75
**AD biomarkers**			
Amyloid PET, Centiloid	67.6 ± 35.5	68.3 ± 46.4	68.9 ± 30.8
Amyloid PET-positive^b^, n (%)	696 (87.9)	158 (79.0)	137 (90.1)
Mesial-temporal tau PET, SUVR	1.51 ± 0.44	1.63 ± 0.23	1.66 ± 0.25
Mesial-temporal tau PET-positive^c^, n (%)	99 (47.1)	180 (84.9)	78 (51.3)
Amyloid PET tracer (^18^F-florbetapir / ^18^F-florbetaben), n (%)	634 (80.1) / 158 (19.9)	125 (62.5) / 75 (37.5)	89 (58.6) / 63 (41.4)

Data are presented as mean ± standard deviation or n (%). A total of 210 participants with estimated amyloid PET positivity age had a concurrent tau PET scan, and 200 participants with estimated tau PET positivity age had a concurrent amyloid PET scan.

a Obesity and hypertension data correspond to measurements obtained within one year of the amyloid PET acquisition. Participants were classified as obese when BMI > 30 kg/m², and as hypertensive when systolic blood pressure ≥ 130 mmHg and diastolic blood pressure ≥ 80 mmHg. Medical history of chronic kidney disease, diabetes, and dyslipidemia was collected at study entry and was available for 499 participants with estimated age at amyloid PET positivity, 68 with estimated age at tau PET positivity, and 46 with both estimates. White matter hyperintensity volume (cm³) was normalized to total intracranial volume and is expressed as a percentage.

b Amyloid PET positivity threshold: > 0.78 SUVR for FBP scans and > 0.74 SUVR for FBB scans

c Mesial-temporal tau PET positivity threshold: > 1.41 SUVR.

Supplementary Table ST1 shows ADNI participant characteristics at the initial PET visit, stratified by their estimated age at biomarker positivity ( < 65, 65-75 or > 75 years). After adjusting for chronological age, participants with a younger amyloid PET positivity age demonstrated significantly worse cognitive performance at their initial PET visit, characterized by higher CDR-SB scores and lower mPACC scores, and were less likely to be CU (as defined by a CDR = 0). However, this younger amyloid PET positivity age group also exhibited lower white matter hyperintensity (WMH) burden and a lower prevalence of chronic kidney disease. Conversely, participants with an older tau PET positivity age were more likely to be CU and had a significantly higher WMH burden at their first PET visit compared to the younger group. The tau PET positivity age groups did not significantly differ regarding other vascular risk factors or baseline cognitive performance measured with CDR-SB or mPACC.

In the HABS replication cohort, 104 participants had an estimated amyloid PET positivity age, 48 had an estimated tau PET positivity age, and 24 had both estimates (Supplementary Table ST2). The mean estimated amyloid and tau PET positivity ages were 66.9 ± 9.6 years and 73.2 ± 9.3 years, respectively. Consistent with findings in ADNI, *APOE*-ε4 carriers exhibited younger estimated amyloid and tau PET positivity ages (Supplementary Fig. S6A, S6B). Although women showed numerically younger estimated positivity ages for both biomarkers, this analysis was underpowered to replicate the sex effect observed in ADNI.

HABS participant characteristics, stratified by their estimated age at biomarker positivity ( < 65, 65-75, and > 75 years), are presented in Supplementary Table ST3. Across the amyloid PET positivity age groups, there was a significant difference in educational attainment, though baseline cognitive performance (measured by CDR-SB and mPACC) did not significantly differ after adjusting for chronological age. Conversely, across the tau PET positivity age groups, baseline mPACC scores significantly differed, with the oldest group ( > 75 years) exhibiting the lowest scores. Notably, after adjusting for chronological age, baseline WMH burden did not significantly differ across any of the biomarker positivity age groups. Information regarding the prevalence of comorbidities was not available in the HABS cohort.

### 3.2 Amyloid-tau time interval distribution and differences by *APOE*-ε4 status and sex

**Fig. 2:**
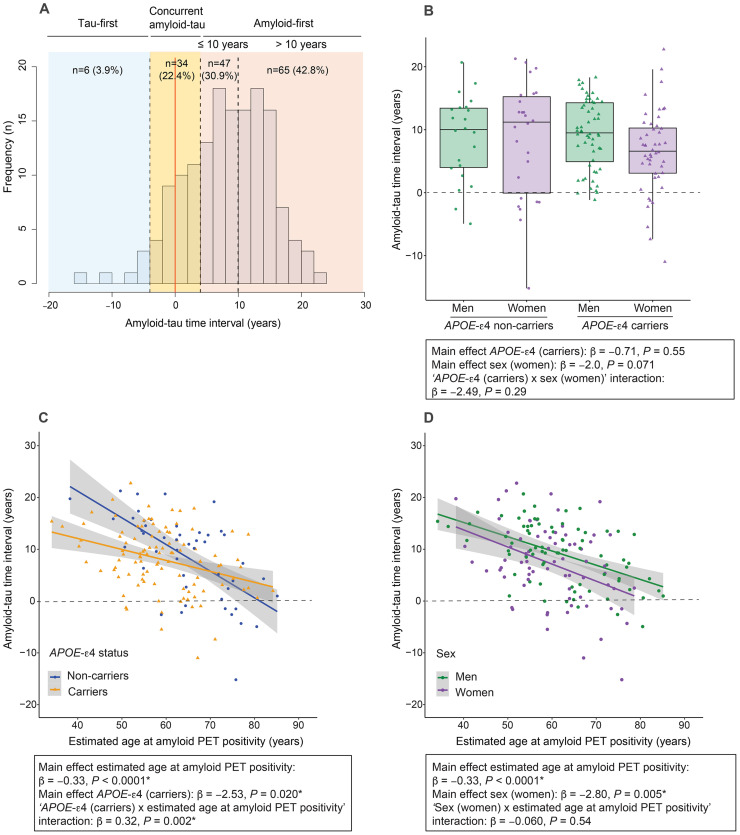
**Characteristics of the amyloid-tau time interval and effect of *APOE*-ε4 status and sex. A.** Distribution of the amyloid-tau time interval. The “concurrent amyloid-tau” group was defined by an interval of ± 4 years, reflecting cumulative mean absolute error for estimated positivity ages (see Supplementary Methods). Participants that became amyloid-positive first and tau-positive 4 to 10 years later were classified as “amyloid first ≤ 10 years”, and participants that became amyloid-positive first and tau-positive more than 10 years later were classified as “amyloid-first > 10 years”. **B.** Amyloid-tau time interval by *APOE*-ε4 status and sex. Box plots depict the median (horizontal bar), interquartile range (IQR; box), and 1.5 × IQR (whiskers). **C, D**. Scatter plots show the association between estimated age at amyloid PET positivity and the amyloid-tau time interval by *APOE*-ε4 status (C) and sex (D). Solid lines indicate the regression line and 95% CIs. The dashed horizontal line at 0 years indicates concurrent amyloid and tau PET positivity. Positive values reflect individuals who became tau PET-positive after amyloid PET positivity; negative values indicate tau PET positivity preceding amyloid PET positivity. Beta coefficients and *P*-values from linear regression models are reported for the main effects of *APOE*-ε4 status (carriers *vs*. non-carriers), sex (women *vs.* men) or estimated age at amyloid PET positivity, and their interaction terms. All models included *APOE*-ε4 status and sex as covariates. Interaction terms were added in separate models.

The mean amyloid-tau time interval in ADNI was 8.2 ± 6.8 years. The amyloid-tau time interval distribution (Fig. 2A) showed that 34 individuals (22.4%) had a “concurrent amyloid-tau” profile (-4 years ≤ interval ≤ 4 years). Most individuals (*n* = 112; 73.7%) showed amyloid PET positivity preceding tau PET positivity (‘amyloid-first’ group), with 65 (58.0%) of them having a time interval exceeding 10 years. Six individuals (3.9%) had tau PET positivity before amyloid PET positivity (‘tau-first’ group).

*APOE*-ε4 status, sex, or their interaction were not significantly associated with the amyloid-tau time interval (Fig. 2B). However, when amyloid PET positivity age was included in the model, an older age at amyloid PET positivity was significantly associated with a shorter amyloid-tau time interval (β = −0.33, *P* < 0.0001; Fig. 2C, D). Of note, sensitivity analysis using baseline chronological age instead of amyloid PET positivity age showed no significant association with the amyloid-tau time interval (β = 0.10; *P* = 0.27).

In the extended model accounting for the estimated amyloid PET positivity age, *APOE*-ε4 carriers had a significantly shorter amyloid-tau time interval compared to non-carriers (β = −2.53, *P* = 0.020; Fig. 2C). Similarly, women had a significantly shorter amyloid-tau time interval compared to men (β = −2.80, *P* = 0.005; Fig. 2D). Furthermore, a significant interaction was observed between *APOE-*ε4 status and estimated age at amyloid PET positivity (*P* = 0.002), indicating that the inverse association between an older amyloid PET positivity age and a shorter amyloid-tau time interval was driven more strongly by *APOE-*ε4 non-carriers (Fig. 2C). The two-way interaction between amyloid PET positivity age and sex was not statistically significant (*P* = 0.54; Fig. 2D), nor was the three-way interaction among amyloid PET positivity age, sex, and *APOE-*ε4 status (*P* = 0.92).

Supplementary Table ST4 shows ADNI participant characteristics at the initial PET visit stratified by their amyloid-tau time interval. There were no statistically significant differences across the groups in chronological baseline age, sex, *APOE-*ε4 status, educational attainment, or ethno-racial background. Baseline cognitive performance (CDR-SB and mPACC) and global CDR scores also did not differ significantly. Among the evaluated vascular risk factors, the prevalence of obesity was significantly higher in the “concurrent amyloid-tau” group (*P* = 0.010). A significant overall difference in total WMH burden was observed across the interval groups (*P* = 0.028); however, post-hoc pairwise comparisons between each pair of groups were not statistically significant. Notably, these findings should be interpreted with caution due to the small sample size of the “tau-first” group (*n* = 6).

In the HABS cohort, the mean amyloid-tau time interval was 6.1 ± 7.6 years, with the majority of participants following either a “concurrent amyloid-tau” (41.7%) or an “amyloid-first” (54.2%) pathway (Supplementary Fig. S7A). Consistent with the ADNI findings, an older estimated amyloid PET positivity age was significantly associated with a shorter amyloid-tau time interval (β = −0.44, *P* = 0.009; Supplementary Fig. S7B, S7C). The independent and interactive effects of sex and *APOE*-ε4 status on the amyloid-tau interval, as well as comparisons across interval groups, could not be evaluated in HABS due to insufficient statistical power.

Finally, to contrast biochemical and radiological disease timeline estimates in ADNI, sensitivity analyses were conducted by modeling the estimated age of plasma p-tau217 positivity using a GMM-derived cutoff of 0.257 pg/mL (Supplementary Methods, Supplementary Fig. S5). The mean estimated age for plasma p-tau217 positivity was 71.4 ± 9.2 years. The mean time interval from amyloid PET positivity to plasma p-tau217 positivity was 8.6 ± 6.6 years, demonstrating a distribution similar to that of the radiological amyloid-tau PET interval (Supplementary Fig. S8). When directly comparing these two intervals within the same subset of individuals (*n* = 107), the amyloid-tau PET interval was nearly identical at 8.6 ± 6.5 years. This indicates a tight temporal alignment between the crossing of the systemic plasma p-tau217 threshold and the emergence of early mesial-temporal tau PET positivity. Given this concordance, the tau PET-based timeline was retained for all subsequent clinical progression analyses.

### 3.3 Effects of biomarker positivity age and amyloid–tau time interval on symptom onset and clinical progression

#### 3.3.1 Association with time to symptom onset

AFT survival models assessed factors influencing the time to symptom onset (defined as progression from a CDR = 0 to CDR > 0) following amyloid or tau PET positivity.

In ADNI, 94 participants with an estimated age at amyloid PET positivity and 32 participants with an estimated age at tau PET positivity progressed from CDR = 0 to CDR > 0 after becoming amyloid or tau PET- positive, respectively. In a basic model including baseline chronological age, sex, *APOE*-ε4 status and educational attainment (Model A1 in Table 2), the time to symptom onset following tau PET positivity was 67% longer for women than for men (Time Ratio [TR] = 1.67; *P* = 0.009; Model A1 in Table 2). This independent sex effect was not significant following amyloid PET positivity (TR = 1.19; *P* = 0.10; Model A1 in Table 2). However, a significant interaction between sex and *APOE-*ε4 status was observed following amyloid PET positivity (TR = 1.64; *P* = 0.024; Model A2 in Table 2), indicating that female *APOE-ε4* carriers had a 64% longer time to symptom onset compared to men *APOE-ε4* carriers. No significant sex by *APOE-*ε4 status interaction was observed following tau PET positivity (TR = 1.32; *P* = 0.51; Model A2 in Table 2). Independently, baseline chronological age, *APOE-*ε4 status, and educational attainment did not significantly influence the time to symptom onset.

**Table 2:** Effects of estimated biomarker positivity age and the amyloid-tau time interval on time to symptom onset.

	**Time to symptom onset after amyloid PET positivity**		**Time to symptom onset after tau PET positivity**	
	**Time ratio [95% CI]**	***P-*****value**	**Time ratio [95% CI]**	***P*****-value**
**A: Effect of estimated biomarker positivity age on time to symptom onset**				
**Model A1:**				
Age (baseline)	0.98 [0.96-1.00]	0.051	0.99 [0.96-1.03]	0.72
*APOE-*ε4 status (carriers)	0.99 [0.81-1.23]	0.96	1.16 [0.80-1.70]	0.43
Sex (women)	1.19 [0.97-1.47]	0.10	1.67 [1.14-2.46]	0.009*
Educational attainment (college)	1.05 [0.76-1.44]	0.78	0.99 [0.42-2.36]	0.99
Educational attainment (postgraduate)	1.24 [0.89-1.74]	0.21	1.28 [0.57-2.87]	0.54
**Model A2: Model A1 + ‘sex x*****APOE-*****ε4 status’ interaction**				
Sex (women) x *APOE-*ε4 status (carriers)	1.64 [1.07-2.53]	0.024*	1.32 [0.58-3.02]	0.51
**Model A3: Model A1 + biomarker positivity age**				
Biomarker positivity age	0.96 [0.95-0.97]	< 0.0001	0.97 [0.95-0.99]	0.002*
*APOE-*ε4 status (carriers)	0.87 [0.75-1.02]	0.085	1.08 [0.78-1.50]	0.63
Sex (women)	1.03 [0.89-1.21]	0.68	1.29 [0.90-1.84]	0.17
Educational attainment (college)	1.02 [0.81-1.28]	0.87	0.77 [0.39-1.53]	0.46
Educational attainment (postgraduate)	1.23 [0.96-1.58]	0.10	1.02 [0.54-1.94]	0.95
**Model A4: Model A3 + ‘biomarker positivity age x*****APOE-*****ε4 status’ interaction**				
Biomarker positivity age x *APOE-*ε4 status (carriers)	1.00 [0.98-1.01]	0.81	1.00 [0.97-1.04]	0.97
**Model A5: Model A3 + ‘biomarker positivity age x sex’ interaction**				
Biomarker positivity age x sex (women)	1.00 [0.98-1.01]	0.60	0.99 [0.95-1.03]	0.48
**Model A6: Model A3 + ‘biomarker positivity age x*****APOE-*****ε4 status x sex’ interaction**				
Biomarker positivity age x sex (women) x *APOE-*ε4 status (carriers)	0.97 [0.93-1.00]	0.081	1.04 [0.95-1.13]	0.42
**B: Effect of the amyloid-tau time interval on time to symptom onset**				
**Model B1**				
Amyloid-tau time interval	1.06 [1.00-1.08]	< 0.0001	0.98 [0.96-1.00]	0.11
Sex (women)	1.34 [1.00-1.72]	0.024*	1.43 [0.94-2.18]	0.093
*APOE-*ε4 status (carriers)	1.14 [0.90-1.45]	0.27	1.11 [0.74-1.66]	0.62
Educational attainment (college)	0.95 [0.60-1.50]	0.83	1.12 [0.54-2.31]	0.76
Educational attainment (postgraduate)	1.03 [0.60-1.63]	0.89	1.14 [0.55-2.39]	0.73
**Model B2: Model B1 + ‘amyloid-tau time interval x sex’ interaction**				
Amyloid-tau time interval x sex (women)	0.95 [0.90-0.99]	0.010*	0.94 [0.89-0.99]	0.028*
**Model B3: Model B1 + ‘amyloid-tau time interval x*****APOE-*****ε4 status’ interaction**				
Amyloid-tau time interval x *APOE-*ε4 status (carriers)	0.98 [0.90-1.01]	0.14	0.98 [0.94-1.03]	0.40
**Model B4: Model B1 + biomarker positivity age**				
Amyloid-tau time interval	1.05 [1.03-1.06]	< 0.0001	0.99 [0.97-1.02]	0.54
Biomarker positivity age	0.98 [0.96-0.99]	0.003*	0.97 [0.94-0.99]	0.013*
Sex (women)	1.17 [0.92-1.50]	0.21	1.14 [0.75-1.74]	0.53
*APOE-*ε4 status (carriers)	1.08 [0.86-1.35]	0.52	1.00 [0.69-1.46]	0.10
Educational attainment (college)	0.77 [0.49-1.20]	0.24	0.82 [0.40-1.70]	0.60
Educational attainment (postgraduate)	0.91 [0.60-1.38]	0.65	0.97 [0.49-1.92]	0.93
**Model B5: Model B4 + ‘amyloid-tau time interval x sex’ interaction**				
Amyloid-tau time interval x sex (women)	0.97 [0.94-1.01]	0.15	0.97 [0.92-1.03]	0.29
**Model B6: Model B4 + ‘amyloid-tau time interval x*****APOE-*****ε4 status’ interaction**				
Amyloid-tau time interval x *APOE-*ε4 status (carriers)	0.98 [0.95-1.01]	0.14	0.98 [0.94-1.02]	0.26

Accelerated Failure Time (AFT) models were used to examine the main effects of the estimated biomarker positivity age (A) or the amyloid-tau time interval (B) along with sex (women *vs.* men), *APOE-*ε4 status (carriers *vs.* non-carriers) and educational attainment (college or postgraduate *vs.* high school or less), on the time from amyloid or tau PET positivity to conversion from CDR = 0 to CDR > 0. Interaction terms with sex or *APOE-*ε4 were added in separate models. All models included sex, *APOE-*ε4 status and educational attainment as covariates. A time ratio > 1 indicates slower progression (longer time to impairment), whereas a time ratio < 1 indicates faster progression (shorter time to impairment).

An older age at biomarker positivity was associated with a shorter time to symptom onset. Specifically, each one-year increase in the estimated age at amyloid or tau PET positivity corresponded to a 4% (TR = 0.96; *P* < 0.0001) or 3% (TR = 0.97; *P* = 0.002) shorter time to symptom onset, respectively (Model A3 in Table 2, Supplementary Fig. S9A, S9B). These effects were not significantly modified by sex, *APOE*-ε4 status, or their three-way interaction (Models A4-A6 in Table 2). Notably, upon adjusting for the estimated age at biomarker positivity, the previously observed independent effect of female sex on delaying symptom onset was no longer significant (Model A1 *vs.* Model A3 in Table 2), suggesting that the observed longer time to symptom onset observed in women was largely driven by their younger age at biomarker positivity.

Next, we examined the effect of the amyloid-tau time interval on the time to symptom onset among the subset of participants with estimated ages for both amyloid and tau PET positivity (30 of whom progressed from CDR = 0 to CDR > 0). A shorter amyloid-tau time interval was associated with a more rapid onset of symptoms; specifically, each one-year decrease in the interval was associated with a 6% shorter time to symptom onset following amyloid PET positivity (TR = 1.06; *P* < 0.0001; Model B1 in Table 2, Supplementary Fig. S9C). This effect remained significant independently of the amyloid PET positivity age (TR = 1.05; *P* < 0.0001; Model B4 in Table 2). Conversely, the duration of amyloid-tau time interval did not significantly influence the time to symptom onset following tau PET positivity (TR = 0.98; *P* = 0.11 and TR = 0.99; *P* = 0.54, respectively, for Models B1 and B4 in Table 2, Supplementary Fig. S9D). Instead, an older age at tau PET positivity remained the only significant predictor of a shorter time to symptom onset (TR = 0.97; *P* = 0.013), irrespective of the preceding amyloid-tau time interval (Model B4 in Table 2). While initial models suggested significant interactions with sex, suggesting an attenuation of the longer interval’s protective effect in women, these interactions became non-significant after adjusting for the age at amyloid or tau PET positivity (Models B2 and B5 in Table 2). *APOE-*ε4 status did not significantly modify these associations (Models B3 and B6 in Table 2), and three-way interactions could not be assessed due to sample size limitations.

In the HABS cohort, 55 participants with an estimated age at amyloid PET positivity, 29 with an estimated age at tau PET positivity, and 17 with both estimates progressed to symptom onset. The significant effect of the estimated age at biomarker positivity on the time to symptom onset was replicated in this cohort: each one-year increase in the estimated age at amyloid or tau PET positivity was associated with a 3% (TR = 0.97; *P* < 0.0001) or 4% (TR = 0.96; *P* = 0.005) shorter time to symptom onset, respectively (Model A2 in Supplementary Table ST5, Supplementary Fig. S10A, Supplementary Fig. S10B). The effect of the amyloid-tau time interval duration on the time to symptom onset following amyloid PET positivity was also replicated in HABS: each one-year decrease in the amyloid-tau time interval was significantly associated with a 4% shorter time to symptom onset following amyloid PET positivity (TR = 1.04; *P* = 0.007; Model B1 in Supplementary Table ST5). This effect remained significant after adjusting for the estimated amyloid PET positivity age (TR = 1.03; *P* = 0.040; Model B2 in Supplementary Table ST5, Supplementary Fig. S10C). Furthermore, consistent with ADNI findings, the amyloid-tau time interval had no significant effect on the time to symptom onset following tau PET positivity (TR = 0.98; *P* = 0.40 and TR = 0.99; *P* = 0.62, respectively, for Models B1 and B2 in Supplementary Table ST5, Supplementary Fig. S10D). Independent effects of sex or *APOE-*ε4 status on the time to symptom onset were not observed in HABS, and their interactions could not be evaluated due to insufficient statistical power.

#### 3.3.2 Association with clinical progression

Next, we assessed differences in the rates of cognitive decline following symptom onset, defined as the first visit with a CDR > 0, across biomarker positivity age groups and amyloid-tau time interval groups in ADNI (see Supplementary Table ST6 for CI participant characteristics).

**Fig. 3:**
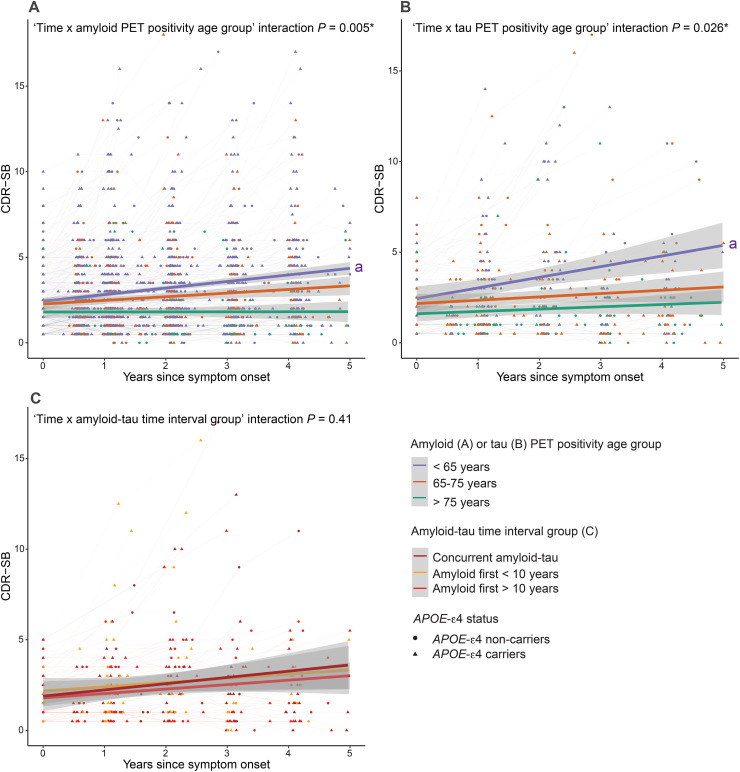
**Clinical progression after symptom onset by estimated biomarker positivity age group or amyloid-tau time interval group**. Linear mixed-effects models with random intercepts and slopes were used to model CDR-SB trajectories as a function of years since symptom onset (defined as the first visit with CDR > 0), grouping individuals by estimated age at amyloid PET positivity **(A)**, tau PET positivity **(B)** or by the amyloid–tau time interval **(C).** Interaction terms between time and estimated biomarker age group or interval group were evaluated to assess differences in the rate of cognitive decline. Pairwise contrasts were conducted to compare group-specific cognitive decline rates (Supplementary Table ST7). Individual trajectories are shown, where each line connects multiple observations from the same individual. In C, the “tau-first” group was excluded from the analysis due to the small sample size (*n* = 6). *Statistically significant (*P* < 0.05); ^a^Statistically significant (*P* < 0.05) *vs.* ‘ > 75 years’ group.

The linear mixed-effects models revealed significant differences in the rates of CDR-SB decline across both amyloid and tau PET positivity age groups (time by amyloid PET positivity age group interaction *P* = 0.005; time by tau PET positivity age group interaction *P* = 0.026; Fig. 3A, B, Supplementary Table ST7). Post-hoc pairwise comparisons indicated that individuals who became amyloid PET-positive before age 65 showed a significantly faster rate of decline compared to those > 75 years (rate of change in CDR-SB/year: 0.96 [95% CI: 0.84-1.09] vs. 0.41 [95% CI: 0.08-0.73]; *P* = 0.005). A similar trajectory was observed when grouping individuals by tau PET positivity age (rate of change in CDR-SB/year: 1.07 [95% CI: 0.74-1.39] for < 65 years group vs. 0.39 [95% CI: 0.03-0.76] for > 75 years group; *P* = 0.020; Supplementary Table ST7). In contrast, the amyloid-tau time interval did not significantly influence the rates of cognitive decline (time by amyloid-tau time interval group interaction *P* = 0.41; Fig. 3C, Supplementary Table ST7).

No significant modifying effects of sex or *APOE*-ε4 status were found in these associations between biomarker positivity age groups or interval groups and the rate of cognitive decline (Supplementary Table ST7).

In sensitivity analyses, participants were classified by their baseline chronological age using the identical cutoffs ( < 65, 67-75 or > 75 years). In this model, we did not observe a significant interaction between baseline chronological age group and time (time by age group interaction *P* = 0.54; Supplementary Fig S11). These findings suggest that the differing rates of decline are specifically associated with the disease-related biomarker timing rather than chronological age.

These longitudinal analyses could not be performed in the HABS cohort due to insufficient sample sizes.

## 4 Discussion

This study investigated the timing between amyloid and tau PET positivity, and their associations with symptom onset and clinical progression, specifically considering *APOE*-ε4 status and sex differences. Our main findings were: 1) *APOE-*ε4 carriers and women were younger when they became amyloid and tau PET-positive; 2) *APOE-*ε4 carriers, women, and individuals with an older estimated age of amyloid PET positivity had a shorter time interval from amyloid to tau PET positivity; 3) individuals who became amyloid and tau PET-positive at older ages had a shorter time until symptom onset but showed slower rates of cognitive decline thereafter; and 4) a shorter time between amyloid and tau PET positivity was associated with a more rapid onset of symptoms, though this effect diminished once tau pathology had emerged. These results contribute to our understanding of the factors influencing heterogeneity in disease progression following amyloid PET positivity, with implications for identifying high-risk individuals for prevention and early therapeutic trials.

Both *APOE-*ε4 carriership and female sex were independently associated with younger estimated ages of amyloid and tau PET positivity, consistent with their established AD vulnerability [[6], [7], [8], [9]]. The sex effect was more pronounced among *APOE-*ε4 carriers for amyloid PET positivity, suggesting that female *APOE-*ε4 carriers may be at greatest risk for the early development of AD pathology. While interactions between sex and *APOE*-ε4 have been reported for tau vulnerability [10,[13], [14], [15]], we did not observe a statistically significant effect in our sample, potentially due to limited statistical power or the use of a mesial-temporal meta-ROI capturing very early tau accumulation, a stage where sex differences may not yet be apparent.

A key aim of our study was to evaluate the time between amyloid and tau PET positivity (*i.e*. the amyloid-tau time interval) and its implications for clinical progression. We found that most participants followed an “amyloid-first” trajectory, consistent with the canonical AD pathway [1,2]. Regarding factors affecting the amyloid-tau time interval, *APOE-*ε4 carriers and women exhibited a significantly shorter interval, indicating a faster progression to tau pathology following amyloid positivity. Combined with their younger estimated ages of biomarker positivity, these findings reinforce their greater vulnerability to AD pathology. Importantly, an older estimated age at amyloid PET positivity was also associated with a shorter amyloid-tau time interval. This may reflect a higher prevalence of co-pathologies with increasing age [[35], [36], [37], [38], [39]] or reduced brain resistance at older ages [40], which could accelerate tau spread following amyloid accumulation.

It is important to emphasize that our tau PET clock was anchored to a mesial-temporal meta-ROI to deliberately capture the initial seeding of tau pathology. This region is uniquely sensitive to the earliest localized stages of tau accumulation, allowing us to estimate the preclinical therapeutic window following amyloid positivity. To contextualize this against later disease stages, we derived a tau clock using a temporo-parietal ROI (approximating Braak stages IV-V) (Supplementary Fig. S12) and evaluated the clinical status of participants at the time they reached their estimated neocortical tau positivity. We found that 73% of these individuals were already clinically symptomatic (CDR > 0) at the time of their estimated temporo-parietal tau positivity. Because our primary objective is to inform early-stage clinical trials targeting the preclinical or prodromal stages of disease, we kept the mesial-temporal tau clock for our analyses. Modeling intervals based on neocortical spread would largely capture the symptomatic phase of the disease, rather than the early prevention window we aimed to characterize. In this line, our sensitivity analyses contrasting radiological and biochemical intervals revealed that the amyloid-plasma p-tau217 time interval was not significantly shorter than the amyloid-tau PET time interval. This finding suggests that crossing the specific threshold for systemic plasma p-tau217 positivity aligns closely with the earliest focal accumulation of tau in mesial-temporal structures, and that strictly localized radiological markers can identify pathological onset on a timeline highly concurrent with systemic biochemical thresholds.

The observation that older biomarker positivity age was associated with accelerated progression in early disease stages was consistent across both biological and clinical domains. Individuals who became amyloid and tau PET-positive at an older age not only exhibited a shorter amyloid-tau time interval but also experienced earlier symptom onset. One might argue that the apparent faster progression in older individuals may partly reflect study design and follow-up constraints. Specifically, shorter remaining observation times and differential survival at study entry in older participants may lead to an apparent accelerated disease progression rather than true biological differences [41]. We addressed this by performing sensitivity analyses using baseline chronological age instead of estimated biomarker positivity age, revealing that the amyloid-tau time interval was not significantly associated with baseline chronological age. In addition, baseline chronological age did not show a significant effect on the time to symptom onset in AFT models.

In contrast to the effect observed on the time to tau PET positivity and symptom onset, individuals with older biomarker positivity ages showed slower rates of cognitive decline following symptom onset compared to those with younger biomarker positivity ages. This finding highlights a potential disconnect between vulnerability to symptom onset and the subsequent rate of clinical decline. One possible explanation is that younger individuals may have greater cognitive reserve or resilience mechanisms that delay the emergence of clinical symptoms despite accumulating pathology; however, once a specific pathological threshold is crossed, their decline accelerates more rapidly. Notably, our finding of steeper cognitive decline in individuals with younger biomarker positivity ages is consistent with previous reports of faster progression in early-onset AD (EOAD) compared to late-onset AD (LOAD) [[42], [43], [44]]. In this regard, it is important to highlight that significant differences in cognitive decline rates were not observed when baseline chronological age groups were used instead of biomarker positivity age groups. These results support the added value of using biomarker-derived age estimates and indicate that the observed associations are driven by the timing of disease-related biomarker changes rather than chronological age alone.

Beyond the effects of the age at biomarker positivity, a shorter time until tau PET positivity following amyloid PET positivity was associated with an earlier onset of symptoms. Conversely, the amyloid-tau time interval duration did not significantly influence the time to symptom onset following tau PET positivity, nor did it influence rates of cognitive decline. These findings support tau pathology as a primary driver of symptom onset in AD [[17], [18], [19], [20]] and suggest that once tau pathology emerges, clinical decline proceeds similarly, regardless of when amyloid PET positivity initially occurred.

Of interest, a longer time until symptom onset following tau PET positivity was observed in women compared to men, while following amyloid PET positivity, this sex effect emerged only within *APOE*-ε4 carriers. However, adjusting for women’s younger estimated ages of biomarker positivity largely explained this apparent female advantage, suggesting that women experience earlier pathological changes but maintain cognitive resilience longer [45,46]. These findings highlight a sex-dependent vulnerability and the importance of considering both sex and biomarker positivity age when assessing disease progression trajectories. In contrast, no differences by sex or *APOE*-ε4 status were found in the effect of biomarker positivity age or the amyloid-tau time interval on rates of cognitive decline after symptom onset, although these analyses were limited by smaller sample sizes and should be further addressed in future studies.

Overall, our findings contribute to a better understanding of the heterogeneity in the amyloid–tau relationship and how it shapes disease progression [4,[47], [48], [49]]. The key contribution of this study is demonstrating that, following amyloid PET positivity, the time until tau PET positivity and symptom onset varies considerably and is strongly influenced by age, sex and *APOE*-ε4 status. Furthermore, once tau pathology emerges, it becomes the primary driver of clinical decline, irrespective of when amyloid initially started to accumulate.

Importantly, our findings are consistent with and complement recent work [50] showing that at symptomatic stages, chronological age is negatively associated with prevalence of tau positivity. This possibly reflects age-related heterogeneity in AD pathophysiology, where younger individuals follow a more tau-driven and aggressive trajectory, while older individuals may exhibit symptoms with less tau involvement or alternative non-AD processes. In line with our results, *APOE*-ε4 carriership and female sex were also associated with younger ages at amyloid and tau positivity in this study [50]. Another recent study [51] evaluated factors influencing the estimated age at tau positivity and, consistent with our results, reported that higher amyloid burden and *APOE-ε4* were associated with a younger estimated age at tau positivity. They also found that an older age at tau PET positivity was associated with a shorter time from tau PET positivity to dementia. However, unlike our results, they observed an association between low education and literacy and earlier tau positivity and did not report sex differences. Several methodological differences may account for these discrepancies, including variations in the biological clock modeling approach, our study’s use of an earlier tau PET meta-ROI to define the tau timeline, and differing clinical outcome definitions. Specifically, our study focused on the symptom onset (CDR > 0) rather than dementia stage, as it represents a more pertinent milestone for preventive and early-intervention AD clinical trials.

This study has several limitations. First, estimated biomarker positivity ages depend on specific threshold definitions and PET tracers, and are subject to measurement uncertainty, which may limit their precision at the individual level. In addition, the amyloid-tau time interval is derived from both estimated amyloid and tau positivity ages and may therefore compound uncertainty from each estimate, potentially reducing its precision. Further methodological refinement and validation are necessary before these approaches can be reliably applied for individual risk stratification. Second, smaller sample sizes for analyses relative to the tau PET timeline and the amyloid-tau interval limited statistical power, particularly for evaluating interaction terms. Third, our definition of symptom onset relied on a binary clinical threshold (the first visit with a CDR > 0). While the CDR is the gold-standard, clinically valid parameter for defining the onset of meaningful functional and cognitive impairment [3,33,34], it may be insensitive to the earliest, subtle cognitive decline in high-reserve cohorts like ADNI. It is possible that statistical change-points in continuous cognitive composites (e.g., ADNI-Mem or PACC) might detect cognitive inflection points earlier than the CDR. However, because our goal was to model progression toward clinically actionable milestones relevant to early-intervention trial design, we prioritized the clinical validity of the CDR over the psychometric sensitivity of continuous composites. Finally, the heterogeneity in the timing of biomarker positivity and subsequent disease progression, which may be influenced by comorbidities, co-pathologies, lifestyle factors, or social determinants of health, warrants further investigation. Our group comparisons in ADNI showed that individuals with older biomarker positivity ages exhibited higher baseline prevalence of chronic kidney disease and cerebrovascular disease burden, as measured by WMH volume. In addition, a significantly higher prevalence of obesity was observed in participants within the “concurrent amyloid-tau” group. Although these factors may contribute to the faster disease progression observed in these groups, this interpretation should be made with caution. Our sample consisted primarily of participants from ADNI 1, 2 and 3, wherein the overall prevalence of vascular risk factors is relatively low. Moreover, information on common comorbidities or vascular risk factors was collected either at study entry or at first PET visit, precluding the robust assessment of their causal effects on longitudinal disease progression. Future studies incorporating more comprehensive and longitudinal assessments of vascular pathology will be essential to better understand their role in disease progression.

Importantly, several of our key findings were replicated in the HABS cohort, supporting the generalizability of our results beyond ADNI. In particular, we confirmed that an older estimated age at biomarker positivity is associated with a faster progression to both tau PET positivity and symptom onset. Additionally, a shorter interval between amyloid and tau PET positivity was also associated with a more rapid progression to symptom onset in this cohort. Together, these findings reinforce the concept that later onset of pathological processes may be linked to a more aggressive disease course towards clinical impairment. However, not all analyses could be replicated due to limited sample sizes and reduced statistical power in the HABS cohort, particularly for the interaction and longitudinal models. Further validation in larger and more diverse cohorts will be essential to confirm these findings and better characterize the factors influencing disease progression trajectories.

In summary, this study evaluated how estimated ages at amyloid and tau PET positivity, and the time interval between these two key events, shape the timing of symptom onset and clinical progression. We also demonstrated how these dynamics differ by *APOE-*ε4 status and sex, as visually summarized in Supplementary Fig. 13. We found that, following amyloid PET positivity, older individuals, *APOE-*ε4 carriers and women progress to tau PET positivity and symptom onset more rapidly. These findings are crucial for predicting “when” an amyloid-positive individual is likely to develop tau pathology and cognitive impairment, thereby optimizing participant selection for AD clinical trials targeting early disease stages. The influence of age, *APOE-*ε4 and sex on these temporal dynamics underscores their importance in modeling disease trajectories and designing tailored preventive and therapeutic interventions.

## Disclosure

Dr. Schindler has served on scientific advisory boards on biomarker testing and education for Eisai and Novo Nordisk and has received speaking fees for presentations on biomarker testing from Eisai, Eli Lilly, and Novo Nordisk. Dr. Tosun reported grants from National Institutes of Health during the conduct of the study.

No Generative AI or AI-assisted technologies were used in the writing process of this manuscript.

## Study funding

Dr. Milà-Alomà receives funding from Alzheimer’s Association Research Fellowship grant program (AARF-23-1141384). This work was supported by National Institutes of Health (NIH) grants (U19 AG024904, R01 AG091657, U01 AG068057, and U24 AG074855) to Dr. Tosun.

## CRediT authorship contribution statement

**Marta Milà-Alomà:** Writing – review & editing, Writing – original draft, Visualization, Validation, Methodology, Investigation, Formal analysis, Data curation, Conceptualization. **Isabella Hausle:** Writing – review & editing, Validation, Methodology, Investigation, Formal analysis, Conceptualization. **Kellen K. Petersen:** Writing – review & editing, Validation, Formal analysis, Conceptualization. **Pamela Thropp:** Writing – review & editing, Project administration, Conceptualization. **Suzanne E. Schindler:** Writing – review & editing, Validation, Methodology, Investigation, Formal analysis, Data curation, Conceptualization. **Duygu Tosun:** Writing – review & editing, Validation, Supervision, Resources, Project administration, Methodology, Funding acquisition, Formal analysis, Data curation, Conceptualization.

## Declaration of competing interest

The authors declare the following financial interests/personal relationships which may be considered as potential competing interests: Dr. Schindler has served on scientific advisory boards on biomarker testing and education for Eisai and Novo Nordisk and has received speaking fees for presentations on biomarker testing from Eisai, Eli Lilly, and Novo Nordisk. Dr. Tosun reported grants from National Institutes of Health during the conduct of the study. If there are other authors, they declare that they have no known competing financial interests or personal relationships that could have appeared to influence the work reported in this paper.

## Appendix Supplementary materials

Image, application 1
