# Supplementary material for: The time interval from amyloid to tau PET positivity varies by age, sex and APOE-ε4 status
Source: J Prev Alzheimers Dis. 2026 Jun 17;13(8):100622. doi: 10.1016/j.tjpad.2026.100622 (PMC13284470; doi:10.1016/j.tjpad.2026.100622)
Supplement: Supplementary file 1 [file mmc1.docx]

**The time interval from amyloid to tau PET positivity varies by age, sex and *APOE*-ε4 status**

Marta Milà-Alomà^1,2^, Isabella Hausle^1^, Kellen K. Petersen^3^, Pamela Thropp^1^, Suzanne E. Schindler^3^, Duygu Tosun^1,2^, for the Alzheimer’s Disease Neuroimaging Initiative*

**Author affiliations**

^1^Northern California Institute for Research and Education, San Francisco, CA, USA

^2^Department of Radiology and Biomedical Imaging, University of California San Francisco, San Francisco, CA, USA

^3^Department of Neurology, Washington University in St. Louis, St. Louis, MO, USA

*Alzheimer’s Disease Neuroimaging Initiative (ADNI): Data used in preparation of this article were obtained from the Alzheimer’s Disease Neuroimaging Initiative (ADNI) database (adni.loni.usc.edu). As such, the investigators within the ADNI contributed to the design and implementation of ADNI and/or provided data but did not participate in analysis or writing of this report. A complete listing of ADNI investigators can be found at: http://adni.loni.usc.edu/wpcontent/uploads/how_to_apply/ADNI_Acknowledgement_List.pdf

**Supplementary Materials**

Table of Contents

[Supplementary Methods 3](#_Toc227945707)

[Amyloid and tau PET clock modelling 3](#_Toc227945708)

[Estimates for the age at amyloid and tau PET positivity 4](#_Toc227945709)

[ADNI plasma p-tau217 clock and temporo-parietal tau PET clock 4](#_Toc227945710)

[References 5](#_Toc227945711)

[Supplementary Table ST1. ADNI participant characteristics by estimated age at biomarker positivity 6](#_Toc227945712)

[Supplementary Table ST2. HABS participant characteristics at first PET acquisition 8](#_Toc227945713)

[Supplementary Table ST3. HABS participant characteristics by estimated age at biomarker positivity 9](#_Toc227945714)

[Supplementary Table ST4. ADNI participant characteristics by amyloid-tau time interval group 11](#_Toc227945715)

[Supplementary Table ST5. Effects of estimated biomarker positivity age and the amyloid-tau time interval on time to symptom onset in HABS 13](#_Toc227945716)

[Supplementary Table ST6. Characteristics of cognitively impaired ADNI participants at first CDR > 0 visit 14](#_Toc227945717)

[Supplementary Table ST7. Rate of decline after symptom onset by biomarker positivity age group or amyloid-tau time interval group 16](#_Toc227945718)

[Supplementary Figure S1. Model fits to estimate amyloid and mesial-temporal tau PET clocks in ADNI 17](#_Toc227945719)

[Supplementary Figure S2. Validation of the amyloid and mesial-temporal tau PET clock estimates in ADNI 18](#_Toc227945720)

[Supplementary Figure S3. Model fits and validation of the amyloid PET clock in HABS 19](#_Toc227945721)

[Supplementary Figure S4. Model fits and validation of the tau PET clock in HABS 20](#_Toc227945722)

[Supplementary Figure S5. Model fits and validation of the plasma p-tau217 clock 21](#_Toc227945723)

[Supplementary Figure S6. Estimated ages at amyloid and tau PET positivity by *APOE*-ε4 status and sex in the HABS 22](#_Toc227945724)

[Supplementary Figure S7. Characteristics of the amyloid-tau time interval in the HABS 23](#_Toc227945725)

[Supplementary Figure S8. Amyloid-plasma p-tau217 time interval distribution 24](#_Toc227945726)

[Supplementary Figure S9. Effect of estimated biomarker positivity age and amyloid-tau time interval on time to symptom onset following amyloid or tau PET positivity in ADNI 25](#_Toc227945727)

[Supplementary Figure S10. Effect of estimated biomarker positivity age and amyloid-tau time interval on time to symptom onset following amyloid or tau PET positivity in HABS 26](#_Toc227945728)

[Supplementary Figure S11. Clinical progression after symptom onset by baseline age group 27](#_Toc227945729)

[Supplementary Figure S12. Model fits and validation of the temporo-parietal tau PET clock 28](#_Toc227945730)

[Supplementary Figure S13. Effects of *APOE*-ε4 status and sex across disease progression milestones 29](#_Toc227945731)

**Supplementary Methods**

## Amyloid and tau PET clock modelling

The modeling approach for amyloid and mesial-temporal tau PET clocks was previously validated in the ADNI cohort^1^. In the current study, this approach was extended to ADNI ^18^F-florbetaben (FBB) amyloid PET data, as well as to ^11^C-Pittsburgh compound B (PiB) amyloid PET and ^18^F-flortaucipir (FTP) tau PET data from the HABS cohort.

In brief, we first determined individual rates of change for both amyloid and tau using linear mixed effect (LME) models. These rates were modeled as a function of the pathology burden at the midpoint of the follow-up period. For amyloid PET, separate LME models were run for ^18^F-florbetapir (FBP), ^18^F-florbetaben (FBB) or ^11^C-Pittsburgh compound B (PiB) data. Generalized Additive Models (GAMs) were then used to model amyloid and tau rates of change as a function of midpoint pathology burden (Supplementary Figures S1A, S1D and S1G for ADNI and Supplementary Figures S3A and S4A for HABS). To ensure optimal model fit and minimize the influence of highly variable data points, a cutoff at the 90th percentile of the variance distribution of fitted values was used to identify and exclude high-variance values (Supplementary Figures S1B, S1E and S1H for ADNI and Supplementary Figures S3B and S4B for HABS). This exclusion resulted in specific intervals for clock modeling: 0.62-1.11 SUVR for FBP amyloid PET, and 0.63-1.19 SUVR for FBB amyloid PET in ADNI (Supplementary Figures S1C and S1F), and 1.0-1.77 DVR for PiB amyloid PET in HABS (Supplementary Figure S3C). For ^18^F-flortaucipir (FTP) mesial-temporal tau PET, the modelled intervals were 0.98-2.04 SUVR for ADNI (Supplementary Figure S1I) and 0.99-1.42 SUVR for HABS (Supplementary Figure S4C). Within these refined SUVR intervals, the time corresponding to each 0.0001 SUVR unit increases was calculated by integrating the inverse of the modeled rates of change.

For the amyloid clock, time 0 was set at a global cortical amyloid PET SUVR of 0.78 for FBP and of 0.74 SUVR for FBB ADNI data. In HABS, time 0 corresponded to a global cortical (frontal, lateral temporal and retrospenial) amyloid PET DVR of 1.20^2,3^. For tau, time 0 was defined at a mesial-temporal meta-ROI tau PET SUVR of 1.41 in ADNI and 1.27 in HABS. Therefore, amyloid or tau time at any given SUVR value was calculated by subtracting the cumulative estimated time at the positivity threshold from the cumulative estimated time at that SUVR value.

To validate our amyloid and tau PET clock models, participants who converted from a negative to a positive PET status during their participation in the ADNI or HABS study were identified (ADNI PET converters: n=81 for FBP amyloid PET, n=14 for FBB amyloid PET, and n= 35 for FTP tau PET; HABS PET converters: 32 for PiB amyloid PET and n=23 for FT tau PET). Their actual age at conversion was calculated by averaging their ages at the last negative scan and at the first positive scan. We then tested the associations between the estimated time from the clock models and actual time since conversion; and estimated time intervals between scans (amyloid or tau time at a scan minus amyloid or tau time at first scan) and the actual time intervals between scans. In ADNI, the estimated amyloid time strongly correlated with actual time since conversion in amyloid PET converters (Spearman’s ρ = 0.74, *P*<0.0001 for FBB amyloid PET and Spearman’s ρ = 0.91 for FBP amyloid PET, *P*<0.0001; Supplementary Figures S2A and S2D). Similarly, the correlation between the estimated tau time and the actual time since conversion in tau PET converters was strong (Spearman’s ρ = 0.76, *P*<0.0001; Supplementary Figure S2G). The correlations between estimated and actual time intervals were also significant (FBB amyloid time: Spearman’s ρ = 0.87, *P*<0.0001, Supplementary Figure S2B; FBP amyloid time: Spearman’s ρ = 0.80, *P*<0.0001, Supplementary Figure S2E; tau time: Spearman’s ρ = 0.58, *P*<0.0001, Supplementary Figure S2H). In HABS, the correlation between estimated time and actual time since conversion was ρ = 0.82 for amyloid time and ρ = 0.66 for tau time (Supplementary Figures S3D and S4D, respectively). The correlation between estimated and actual time intervals were ρ = 0.41 for amyloid and ρ = -0.21 for tau (Supplementary Figures S3E and S4E, respectively).

## Estimates for the age at amyloid and tau PET positivity

Age at amyloid PET positivity was estimated for all individuals with at least one amyloid positive scan by subtracting the corresponding amyloid time from their age at the positive scan. For participants with multiple positive scans, estimated ages were averaged.

In ADNI amyloid PET converters, the correlation between the estimated age at amyloid PET positivity and actual age at conversion was high (FBB amyloid PET: Spearman’s ρ = 0.76, *P*<0.0001; FBP amyloid PET: Spearman’s ρ = 0.96, *P*<0.0001; Supplementary Figures S2C and S2F). The mean absolute error (MAE) for estimated amyloid age was 2.42 years for FBB amyloid PET and 1.63 years for FBP amyloid PET. Age at tau PET positivity was estimated for all tau PET scans in participants with at least one positive tau PET scan. The correlation between estimated age at tau PET positivity and actual age at conversion in tau PET converters was high (Spearman’s ρ = 0.96, *P*<0.0001; Supplementary Figure S2I), with a MAE of 2.3 years. Additionally, the test-retest reliability of the estimated ages at amyloid and tau PET positivity were assessed in ADNI using the median slope of the estimated ages at positivity across SUVR from different positive scans in individuals with multiple positive scans. Median slopes were -0.23 (IQR: -1.30 to 5.03) for FBB amyloid PET age, -0.29 (IQR: -0.52 to 0.09) for FBP amyloid PET age, and -0.52 (IQR: -1.16 to -0.17) for tau PET age.

In HABS PET converters, the correlation between the estimated age at PET positivity and actual age at conversion was ρ = 0.93, *P*<0.0001 for amyloid PET positivity and ρ = 0.88, *P*<0.0001 for tau PET positivity (Supplementary Figures S3F and S4F). The MAEs were 1.95 years for the estimated amyloid PET age and 2.47 years for the estimated tau PET age.

## ADNI plasma p-tau217 clock and temporo-parietal tau PET clock

As sensitivity analyses, in ADNI, a plasma p-tau217 (Fujirebio Lumipulse p-tau217) clock^4^ and a temporo-parietal tau PET clock were estimated using the same approach described above.

For the plasma p-tau217 clock, the interval used for the modelling after exclusion of high variance values was 0.052-0.96 pg/mL (Supplementary Figure S5A-C), and a Gaussian mixture model (GMM)-derived positivity cutoff of 0.257 pg/mL was set as time 0. Plasma p-tau217 time model was validated in the group of individuals that converted from a negative to a positive plasma p-tau217 value throughout the study duration (n=67). The correlation between estimated plasma p-tau217 time with actual time since conversion in plasma p-tau217 converters was Spearman’s ρ = 0.77, *P*<0.0001, Supplementary Figure S5D), and the correlation between estimated and actual time intervals were Spearman’s ρ = 0.40, *P*<0.0001 (Supplementary Figure S5E). In plasma p-tau217 converters, the correlation between the estimated age at plasma p-tau217 positivity and actual age at conversion was Spearman’s ρ = 0.89, *P*<0.0001; Supplementary Figure S5F). The mean absolute error (MAE) for estimated plasma p-tau217 age was 2.64 years. The median slope of the estimated ages at positivity across different positive plasma measures in individuals with multiple positive plasma measures was -0.40 (IQR: -0.86 to 0.08).

The temporo-parietal tau PET clock comprised a FTP tau PET meta-ROI that included banks of superior temporal, cuneus, inferior–superior parietal, inferior-middle-superior temporal, istmuscingulate, lateral occipital, lingal, posterior cingulate, precuneus, and supramarginal. The interval used for the modelling after exclusion of high variance values was 0.99-2.03 SUVR (Supplementary Figure S11A-C). A GMM-derived positivity cutoff of 1.35 SUVR was set as time 0. The temporo-parietal tau PET time model was validated in the group of individuals that converted from a negative to a positive scan throughout the study duration (n=35). The correlation between estimated tau time with actual time since conversion in tau PET converters was Spearman’s ρ = 0.81, *P*<0.0001, Supplementary Figure S11D), and the correlation between estimated and actual time intervals were Spearman’s ρ = 0.50, *P*<0.0001 (Supplementary Figure S11E). In tau PET converters, the correlation between the estimated age at tau PET positivity and actual age at conversion was Spearman’s ρ = 0.95, *P*<0.0001; Supplementary Figure S11F). The mean absolute error (MAE) for estimated tau PET age was 1.77 years. The median slope of the estimated ages at positivity across different positive scans in individuals with multiple positive scans was -0.58 (IQR: -0.72 to -0.28).

## References

1. Milà-Alomà M, Tosun D, Schindler SE, et al. Timing of Changes in Alzheimer’s Disease Plasma Biomarkers as Assessed by Amyloid and Tau PET Clocks. *Annals of Neurology*. 2025;98(3):508-523. doi:10.1002/ana.27285

2. Dagley A, LaPoint M, Huijbers W, et al. Harvard Aging Brain Study: Dataset and accessibility. *Neuroimage*. 2017;144(Pt B):255-258. doi:10.1016/j.neuroimage.2015.03.069

3. Mormino EC, Betensky RA, Hedden T, et al. Amyloid and APOE ε4 interact to influence short-term decline in preclinical Alzheimer disease. *Neurology*. 2014;82(20):1760-1767. doi:10.1212/WNL.0000000000000431

4. Petersen KK, Milà-Alomà M, Li Y, et al. Predicting onset of symptomatic Alzheimerʼs disease with plasma p-tau217 clocks. *Nat Med*. 2026:1-10. doi:10.1038/s41591-026-04206-y

# **Supplementary Table ST1. ADNI participant characteristics by estimated age at biomarker positivity**

|  | **Participant characteristics by estimated age at biomarker positivity** | | | | | | | | | | |
| --- | --- | --- | --- | --- | --- | --- | --- | --- | --- | --- | --- |
|  | **Estimated age at amyloid PET positivity** | | | | | | | **Estimated age at tau PET positivity** | | | |
|  | **< 65**  (n=467; 59.0%) | **65 - 75** (n=225; 28.4%) | **> 75**  (n=100; 12.6%) | | ***P*-value** | | **< 65** (n=68; 32.1%) | | **65 - 75**  (n=81; 38.2%) | **> 75**  (n=63; 29.7%) | ***P*-value** |
| Age, years | 71.6 ± 6.39 | 76.9 ± 6.63 | 81.5 ± 5.39 | | <0.0001* | | 68.6 ± 4.67 | | 76.4 ± 5.29 | 83.9 ± 4.61 | <0.0001* |
| Women, n (%) | 253 (54.2) | 108 (48.0) | 38 (38.0) | | 0.009* | | 41 (60.3) | | 36 (44.4) | 26 (41.3) | 0.059 |
| *APOE*-ε4 carriers, n (%) | 344 (43.4) | 113 (50.2) | 36 (36.0) | | <0.0001* | | 55 (80.9) | | 47 (58.0) | 25 (39.7) | <0.0001* |
| Educational attainment  (High school or less / College / Postgraduate), n (%) | 73 (15.6) / 213 (45.6) / 181 (38.8) | 33 (14.7) / 86 (38.2) / 106 (47.1) | 14 (14.0) / 51 (51.0) / 35 (35.0) | | 0.15 | | 6 (8.8) / 40 (58.8) / 22 (32.4) | | 10 (12.3) / 34 (42.0) / 37 (45.7) | 4 (6.3) / 29 (46.0) / 30 (47.6) | 0.21 |
| Ethno-racial background (Black / White / Other), n (%) | 26 (5.6) / 424 (90.8) / 17 (3.6) | 8 (3.6) / 211 (93.8) / 6 (2.7) | 5 (5.0) / 93 (93.0) / 2 (2.0) | | 0.67 | | 6 (8.8) / 59 (86.8) / 3 (4.4) | | 0 (0.0) / 75 (92.6) / 6 (7.4) | 2 (3.2) / 55 (87.3) / 6 (9.5) | 0.06 |
| Estimated age at amyloid PET positivity, years | 55.8 ± 6.26 | 69.6 ± 3.08 | 79.7 ± 3.90 | | <0.0001* | | 53.1 ± 8.36 | | 60.4 ± 6.50 | 71.6 ± 6.46 | <0.0001* |
| Estimated age at tau PET positivity, years | 65.1 ± 7.68 | 75.7 ± 7.39 | 80.4 ± 7.22 | | <0.0001* | | 58.8 ± 4.96 | | 70.1 ± 2.94 | 81.5 ± 4.66 | <0.0001* |
| CDR-SB | 2.05 ± 2.24 | 1.47 ± 1.90 | 0.95 ± 1.63 | | <0.0001* | | 1.88 ± 1.70 | | 2.02 ± 2.77 | 1.51 ± 2.02 | 0.38 |
| mPACC | -7.69 ± 7.30 | -5.25 ± 6.79 | -4.01 ± 5.68 | | <0.0001* | | -8.36 ± 7.12 | | -7.19 ± 8.26 | -5.60 ± 6.22 | 0.074 |
| CDR (0 / 0.5 / >0.5) at baseline, n (%) | 108 (23.1) / 295 (63.2) / 64 (13.7) | 82 (36.4) / 121 (53.8) / 22 (9.8) | 58 (58.0) / 37 (37.0) / 5 (5.0) | | <0.0001* | | 15 (22.1) / 47 (69.1) / 6 (8.8) | | 31 (38.3) / 36 (44.4) / 14 (17.3) | 26 (41.3) / 30 (47.6) / 7 (11.1) | 0.023* |
| CDR (0 / 0.5 / >0.5) at last visit, n (%) | 86 (18.4) / 257 (55.0) / 124 (26.6) | 84 (37.3) / 97 (43.1) / 42 (18.6) | 48 (48.0) / 43 (43.0) / 9 (9.0) | | <0.0001* | | 14 (20.6) / 38 (55.9) / 16 (23.5) | | 20 (24.7) / 44 (54.3) / 17 (21.0) | 23 (36.5) / 32 (50.8) / 8 (12.7) | 0.18 |
| Number of observations | 4.8 ± 2.9 | 5.8 ± 3.4 | | 6.9 ± 4.04 | | 0.28 | | 3.8 ± 2.8 | 5.4 ± 3.7 | 7.7 ± 4.4 | 0.31 |
| Vascular risk factors^a^ | | | | | | | | | | | |
| Diabetes, n (%) | 23 (8.2) | 20 (13.2) | | 4 (6.1) | | 0.15 | | 0 (0.0) | 4 (16.7) | 1 (2.8) | 0.091 |
| Hypertension, n (%) | 192 (41.1) | 100 (44.4) | | 43 (43.0) | | 0.70 | | 26 (38.2) | 33 (40.7) | 33 (52.4) | 0.22 |
| Obesity, n (%) | 80 (17.2) | 45 (20.2) | | 21 (21.0) | | 0.50 | | 11 (16.4) | 15 (19.0) | 10 (15.9) | 0.87 |
| Dyslipidemia, n (%) | 98 (34.9) | 61 (40.1) | | 20 (30.3) | | 0.33 | | 1 (12.5) | 9 (37.5) | 16 (44.4) | 0.24 |
| Chronic kidney disease, n (%) | 54 (19.2) | 50 (32.9) | | 22 (33.3) | | 0.002* | | 1 (12.5) | 1 (4.2) | 9 (25.0) | 0.10 |
| Total white matter hyperintensity volume, % | 0.42 ± 0.63 | 0.55 ± 0.77 | | 0.70 ± 0.88 | | 0.0007* | | 0.31 ± 0.40 | 0.34 ± 0.41 | 0.93 ± 1.36 | <0.0001* |

Participant characteristics by age at biomarker positivity group. Data correspond to the first PET acquisition visit, unless otherwise specified. Data is shown as mean ± standard deviation or n (%). Differences between groups were tested using analyses of variance (ANOVA) for continuous variables and Chi-square test for categorical variables. Differences in cognitive scores and white matter hyperintensity burden were tested using analyses of covariance (ANCOVA) adjusted by age. ^a^Obesity and hypertension data correspond to measurements obtained within one year of the amyloid PET acquisition. Participants were classified as obese when BMI > 30 kg/m², and as hypertensive when systolic blood pressure ≥ 130 mmHg and diastolic blood pressure ≥ 80 mmHg. Medical history of chronic kidney disease, diabetes, and dyslipidemia was collected at study entry and was available for 499 participants with estimated age at amyloid PET positivity (n=281 in <65; n=152 in 65-75; n=66 in >75), and for 68 with estimated age at tau PET positivity (n=8 in <65; n=24 in 65-75; n=36 in >75). White matter hyperintensity volume (cm³) was normalized to total intracranial volume and is expressed as a percentage. *Statistically significant (*P*<0.05)

# **Supplementary Table ST2. HABS participant characteristics at first PET acquisition**

|  | **Cohort with estimated age at amyloid PET positivity (N=104)** | **Cohort with estimated age at tau PET positivity (N=48)** | **Cohort with PET positivity age for both amyloid and tau (N=24)** |
| --- | --- | --- | --- |
| Age, years | 74.9 ± 6.45 | 76.4 ± 8.30 | 74.8 ± 7.68 |
| Women, n (%) | 64 (61.5) | 28 (58.3) | 16 (66.7) |
| *APOE*-ε4 carriers, n (%) | 51 (49.0) | 17 (35.4) | 13 (54.2) |
| Educational attainment  (High school or less / College / Postgraduate), n (%) | 21 (20.2) / 38 (36.5) / 45 (43.3) | 8 (16.7) / 17 (35.4) / 23 (47.9) | 4 (16.7) / 6 (25.0) / 14 (58.3) |
| Ethno-racial background (Black / White / Other), n (%) | 9 (8.7) / 93 (89.4) / 2 (1.9) | 8 (16.7) / 39 (81.2) / 1 (2.1) | 3 (12.5) / 21 (87.5) / 0 (0.0) |
| CDR 0 / 0.5 / >0.5, n (%) | 103 (99.0) / 1 (1.0) / 0 (0.0) | 43 (87.6) / 5 (10.4) / 0 (0.0) | 21 (87.5) / 3 (12.5) / 0 (0.0) |
| CDR-SB | 0.10 ± 0.23 | 0.19 ± 0.30 | 0.21 ± 0.33 |
| mPACC | -0.02 ± 0.67 | 0.08 ± 0.85 | 0.05 ± 0.79 |
| Vascular risk factors | | | |
| Total white matter hyperintensity volume^a^, % | 0.35 ± 0.57 | 0.42 ± 0.66 | 0.48 ± 0.89 |
| AD biomarkers | | | |
| Amyloid PET, Centiloid | 49.19 ± 28.2 | 37.9 ± 35.7 | 60.61 ± 31.5 |
| Amyloid PET-positive^b^, n (%) | 76 (73.1) | 22 (45.8) | 21 (87.5) |
| Mesial-temporal tau PET, SUVR | 1.18 ± 0.12 | 1.27 ± 0.08 | 1.30 ± 0.08 |
| Mesial-temporal tau PET-positive^c^, n (%) | 2 (14.3) | 26 (54.2) | 18 (75.0) |

Data are presented as mean ± standard deviation or n (%). 24 participants had estimates for both amyloid and tau PET positivity age and overlapped between the two cohorts. Only 14 participants with estimated amyloid PET positivity age had a concurrent (less than a year) tau PET scan. All participants with estimated tau PET positivity age had a concurrent amyloid PET scan. ^a^White matter hyperintensity volume (cm³) was normalized to total intracranial volume and is expressed as a percentage ^b^Amyloid PET positivity threshold > 1.20 DVR. ^c^Mesial-temporal tau PET positivity threshold: > 1.27 SUVR.

# **Supplementary Table ST3. HABS participant characteristics by estimated age at biomarker positivity**

|  | **Participant characteristics by estimated age at biomarker positivity** | | | | | | | |
| --- | --- | --- | --- | --- | --- | --- | --- | --- |
|  | **Estimated age at amyloid PET positivity** | | | | **Estimated age at tau PET positivity** | | | |
|  | **< 65**  (n=42; 40.4%) | **65 - 75** (n=42; 40.4%) | **> 75**  (n=20; 19.2%) | ***P*-value** | **< 65** (n=8; 16.7%) | **65 - 75**  (n=17; 35.4%) | **> 75**  (n=23; 47.9%) | ***P*-value** |
| Age, years | 71.9 ± 5.65 | 75.8 ± 5.78 | 79.5 ± 6.31 | <0.0001* | 64.3 ± 6.91 | 74.2 ± 4.24 | 82.2 ± 5.17 | <0.0001* |
| Women, n (%) | 27 (64.3) | 25 (59.5) | 12 (60.0) | 0.89 | 6 (75.0) | 11 (64.7) | 11 (47.8) | 0.33 |
| *APOE*-ε4 carriers, n (%) | 28 (66.7) | 22 (52.4) | 1 (5.0) | <0.0001* | 4 (50.0) | 9 (52.9) | 4 (17.4) | 0.043* |
| Educational attainment  (High school or less / College / Postgraduate), n (%) | 5 (11.9) / 14 (33.3) / 23 (54.8) | 8 (19.0) / 20 (47.6) / 14 (33.3) | 8 (40.0) / 4 (20.0) / 8 (40.0) | 0.031* | 1 (12.5) / 4 (50.0) / 3 (37.5) | 1 (5.9) / 6 (35.3) / 10 (58.8) | 6 (26.1) / 7 (30.4) / 10 (43.5) | 0.43 |
| Ethno-racial background (Black / White / Other), n (%) | 2 (4.8) / 39 (92.9) / 1 (2.4) | 6 (14.3) / 35 (83.3) / 1 (2.4) | 1 (5.0) / 19 (95.0) / 0 (0.0) | 0.50 | 2 (25.0) / 6 (75.0) / 0 (0.0) | 3 (17.6) / 14 (82.4) / 0 (0.0) | 3 (13.0) / 19 (82.6) / 1 (4.3) | 0.80 |
| Estimated age at amyloid PET positivity, years | 57.3 ± 4.32 | 70.0 ± 2.77 | 80.5 ± 4.20 | <0.0001* | 55.2 ± 4.83 | 62.7 ± 8.16 | 71.3 ± 9.44 | 0.007* |
| Estimated age at tau PET positivity, years | 65.7 ± 8.46 | 74.0 ± 6.27 | 78.8 ± 5.25 | 0.014* | 58.2 ± 4.25 | 69.4 ± 3.01 | 81.1 ± 3.68 | <0.0001* |
| CDR-SB | 0.12 ± 0.29 | 0.08 ± 0.19 | 0.07 ± 0.18 | 0.71 | 0.00 ± 0.00 | 0.18 ± 0.30 | 0.27 ± 0.34 | 0.091 |
| PACC | 0.06 ± 0.61 | -0.06 ± 0.73 | -0.13 ± 0.68 | 0.55 | 0.39 ± 0.54 | 0.42 ± 0.68 | -0.28 ± 0.92 | 0.015* |
| CDR (0 / 0.5 / >0.5) at baseline, n (%) | 41 (97.6) / 1 (2.4) / 0 (0.0) | 42 (100.0) / 0 (0.0) / 0 (0.0) | 20 (100.0) / 0 (0.0) / 0 (0.0) | 0.48 | 8 (100.0) / 0 (0.0) / 0 (0.0) | 15 (88.2) / 2 (11.8) / 0 (0.0) | 20 (87.0) / 3 (13.0) / 0 (0.0) | 0.55 |
| CDR (0 / 0.5 / >0.5) at last visit, n (%) | 33 (78.6) / 8 (19.0) / 1 (2.4) | 31 (73.8) / 9 (21.4) / 2 (4.8) | 18 (90.0) / 2 (10.0) / 0 (0.0) | 0.63 | 6 (75.0) / 2 (25.0) / 0 (0.0) | 14 (82.4) / 3 (17.6) / 0 (0.0) | 15 (65.3) / 7 (30.4) / 1 (4.3) | 0.59 |
| Number of observations | 8.43 ± 2.89 | 8.79 ± 3.05 | 7.75 ± 3.61 | 0.61 | 7.5 ± 1.41 | 9.88 ± 2.15 | 10.0 ± 2.44 | 0.015* |
| Vascular risk factors^a^ | | | | | | | | |
| Total white matter hyperintensity volume^a^, % | 0.39 ± 0.75 | 0.37 ± 0.49 | 0.24 ± 0.17 | 0.62 | 0.22 ± 0.28 | 0.26 ± 0.25 | 0.61 ± 0.89 | 0.18 |

Participant characteristics by age at biomarker positivity group. Data correspond to the first PET acquisition visit, unless otherwise specified. Data is shown as mean ± standard deviation or n (%). Differences between groups were tested using analyses of variance (ANOVA) for continuous variables and Chi-square test for categorical variables. Differences in cognitive scores and white matter hyperintensity burden were tested using analyses of covariance (ANCOVA) adjusted by age.

^a^White matter hyperintensity volume (cm³) was normalized to total intracranial volume and is expressed as a percentage. *Statistically significant (*P*<0.05)

# **Supplementary Table ST4. ADNI participant characteristics by amyloid-tau time interval group**

|  | **Participant characteristics by amyloid-tau time interval group** | | | | |
| --- | --- | --- | --- | --- | --- |
|  | **Tau-first**  (n=6; 3.9%) | **Concurrent amyloid-tau**  (n=34; 22.4%) | **Amyloid-first**  (n=112; 73.7%) | | ***P*-value** |
|  |  |  | **< 10 years (n=47; 42.0%)** | **> 10 years**  **(n=65; 58.0%)** |  |
| Age, years | 71.7 ± 3.37 | 72.0 ± 7.39 | 72.8 ± 6.19 | 74.4 ± 6.49 | 0.45 |
| Women, n (%) | 5 (83.3) | 16 (47.1) | 24 (51.1) | 29 (44.6) | 0.33 |
| *APOE*-ε4 carriers, n (%) | 3 (50.0) | 23 (67.6) | 39 (83.0) | 39 (60.0) | 0.05 |
| Educational attainment  (High school or less / College / Postgraduate), n (%) | 0 (0.0) / 1 (16.7) / 5 (83.3) | 3 (8.8) / 16 (47.1) / 15 (44.1) | 3 (6.4) / 28 (59.6) / 16 (34.0) | 11 (16.9) / 29 (44.6) / 25 (38.5) | 0.13 |
| Ethno-racial background (Black / White / Other), n (%) | 0 (0.0) / 6 (100.0) / 0 (0.0) | 2 (5.9) / 31 (91.2) / 1 (2.9) | 4 (8.5) / 40 (85.1) / 3 (6.4) | 0 (0.0) / 59 (90.8) / 6 (9.2) | 0.27 |
| CDR-SB | 0.83 ± 0.82 | 1.31 ± 1.44 | 1.37 ± 1.42 | 1.03 ± 1.60 | 0.53 |
| mPACC | -4.26 ± 4.67 | -6.08 ± 6.25 | -6.77 ± 6.03 | -4.46 ± 6.16 | 0.29 |
| CDR (0 / 0.5 / >0.5) at baseline, n (%) | 2 (33.3) / 4 (66.7) / 0 (0.0) | 11 (32.3) / 21 (61.8) / 2 (5.8) | 13 (27.7) / 32 (68.0) / 2 (4.3) | 29 (44.6) / 33 (50.8) / 3 (4.6) | 0.52 |
| CDR (0 / 0.5 / >0.5) at last visit, n (%) | 2 (33.3) / 4 (66.7) / 0 (0.0) | 6 (17.6) / 20 (58.8) / 8 (23.5) | 10 (21.3) / 29 (61.7) / 8 (17.0) | 15 (23.1) / 39 (60.0) / 11 (16.9) | 0.85 |
| Estimated age at amyloid PET positivity, years | 71.7 ± 7.82 | 66.7 ± 9.36 | 60.2 ± 9.52 | 57.3 ± 9.35 | <0.0001* |
| Estimated age at tau PET positivity, years | 63.7 ± 8.85 | 67.4 ± 9.85 | 67.4 ± 9.34 | 71.6 ± 9.10 | 0.027* |
| Number of observations | 7.1 ± 4.4 | 6.3 ± 4.1 | 4.8 ± 3.6 | 5.2 ± 3.2 | 0.72 |
| Vascular risk factors^a^ | | | | | |
| Diabetes, n (%) | 0 (0.0) | 4 (30.8) | 1 (7.1) | 0 (0.0) | 0.050 |
| Hypertension, n (%) | 4 (66.7) | 13 (38.2) | 26 (55.3) | 29 (44.6) | 0.33 |
| Obesity, n (%) | 0 (0.0) | 13 (39.4) | 5 (10.6) | 14 (21.5) | 0.010* |
| Dyslipidemia, n (%) | 0 (0.0) | 5 (38.5) | 5 (35.7) | 5 (31.2) | 0.63 |
| Chronic kidney disease, n (%) | 0 (0.0) | 3 (23.1) | 3 (21.4) | 1 (6.2) | 0.46 |
| Total white matter hyperintensity volume, % | 0.23 ± 0.25 | 0.31 ± 0.41 | 0.64 ± 1.18 | 0.36 ± 0.45 | 0.028* |

Participant characteristics by amyloid-tau time interval group. Data correspond to the first PET acquisition visit, unless otherwise specified. Data is shown as mean ± standard deviation or n (%). Differences between groups were tested using analyses of variance (ANOVA) for continuous variables and Chi-square test for categorical variables. Differences in cognitive scores and white matter hyperintensity burden were tested using analyses of covariance (ANCOVA) adjusted by age.

^a^Obesity and hypertension data correspond to measurements obtained within one year of the amyloid PET acquisition. Participants were classified as obese when BMI > 30 kg/m², and as hypertensive when systolic blood pressure ≥ 130 mmHg and diastolic blood pressure ≥ 80 mmHg. Medical history of chronic kidney disease, diabetes, and dyslipidemia was collected at study entry and was available for 46 participants with estimated amyloid-tau interval (n=3 in Tau-first; n=13 in Concurrent amyloid-tau; n=14 in Amyloid-first <10 years; n=16 in Amyloid-first >10 years). White matter hyperintensity volume (cm³) was normalized to total intracranial volume and is expressed as a percentage. *Statistically significant (*P*<0.05).

# **Supplementary Table ST5. Effects of estimated biomarker positivity age and the amyloid-tau time interval on time to symptom onset in HABS**

|  | **Time to symptom onset after amyloid PET positivity** | | **Time to symptom onset after tau PET positivity** | |
| --- | --- | --- | --- | --- |
|  | **Time ratio**  **[95% CI]** | ***P-*value** | **Time ratio [95%CI]** | ***P*-value** |
| **A: Effect of estimated biomarker positivity age on time to symptom onset** | | | | |
| **Model A1:** |  |  |  |  |
| *APOE-*ε4 status (carriers) | 1.11 [0.85-1.45] | 0.45 | 0.96 [0.53-1.77] | 0.91 |
| Sex (women) | 0.89 [0.68-1.18] | 0.42 | 1.03 [0.57-1.87] | 0.91 |
| Educational attainment (college) | 1.20 [0.81-1.78] | 0.36 | 1.45 [0.59-3.55] | 0.41 |
| Educational attainment (postgraduate) | 1.11 [0.79-1.57] | 0.55 | 1.08 [0.50-2.33] | 0.85 |
| **Model A2: Model A1 + biomarker positivity age** |  |  |  |  |
| Biomarker positivity age | 0.97 [0.95-0.98] | **<0.0001*** | 0.96 [0.93-0.99] | **0.005*** |
| *APOE-*ε4 status (carriers) | 0.98 [0.77-1.24] | 0.86 | 0.93 [0.54-1.61] | 0.80 |
| Sex (women) | 0.85 [0.68-1.07] | 0.18 | 0.78 [0.42-1.36] | 0.35 |
| Educational attainment (college) | 1.02 [0.87-1.65] | 0.26 | 1.25 [0.58-2.67] | 0.57 |
| Educational attainment (postgraduate) | 1.10 [0.83-1.46] | 0.51 | 1.08 [0.54-2.18] | 0.83 |
| **B: Effect of amyloid-tau time interval on time to symptom onset** | | | | |
| **Model B1** |  |  |  |  |
| Amyloid-tau time interval | 1.04 [1.01-1.06] | **0.007*** | 0.98 [0.95-1.02] | 0.40 |
| Sex (women) | 1.03 [0.69-1.52] | 0.89 | 1.18 [0.63-2.22] | 0.60 |
| *APOE-*ε4 status (carriers) | 0.96 [0.60-1.53] | 0.85 | 0.68 [0.28-1.62] | 0.38 |
| Educational attainment (college) | 0.99 [0.49-2.05] | 0.99 | 1.48 [0.45-4.85] | 0.52 |
| Educational attainment (postgraduate) | 0.98 [0.57-1.68] | 0.95 | 1.24 [0.50-3.08] | 0.64 |
| **Model B2: Model B1 + biomarker positivity age** |  |  |  |  |
| Amyloid-tau time interval | 1.03 [1.00-1.05] | **0.040*** | 0.99 [0.95-1.03] | 0.62 |
| Biomarker positivity age | 0.98 [0.95-1.00] | **0.016*** | 0.97 [0.94-1.00] | 0.090 |
| Sex (women) | 0.79 [0.52-1.21] | 0.29 | 0.83 [0.40-1.72] | 0.62 |
| *APOE-*ε4 status (carriers) | 1.04 [0.67-1.62] | 0.86 | 0.80 [0.34-1.88] | 0.61 |
| Educational attainment (college) | 0.91 [0.49-1.69] | 0.77 | 1.15 [0.39-3.39] | 0.80 |
| Educational attainment (postgraduate) | 1.04 [0.65-1.67] | 0.88 | 1.16 [0.51-2.64] | 0.72 |

Accelerated Failure Time (AFT) models were used to examine the main effects of the estimated biomarker positivity age (A) or the amyloid-tau time interval (B) along with sex, *APOE-*ε4 status and educational attainment, on the time from amyloid or tau PET positivity to conversion from CDR = 0 to CDR > 0. All models included sex, *APOE-*ε4 status and educational attainment as covariates. A time ratio > 1 indicates slower progression (longer time to impairment), whereas a time ratio < 1 indicates faster progression (shorter time to impairment). Interactions with sex and/or *APOE*-ε4 status could not be evaluated due to sample size limitations.

# **Supplementary Table ST6. Characteristics of cognitively impaired ADNI participants at first CDR > 0 visit**

|  | **Cohort with estimated age at amyloid PET positivity (N=482)** | **Cohort with estimated age at tau PET positivity (N=131)** | **Cohort with estimated PET positivity age for both amyloid and tau (N=96)** |
| --- | --- | --- | --- |
| Age, years | 74.7 ± 7.2 | 74.9 ± 7.1 | 74.6 ± 6.9 |
| Women, n (%) | 217 (45.0) | 56 (42.7) | 43 (44.8) |
| *APOE*-ε4 carriers, n (%) | 315 (65.4) | 73 (55.7) | 60 (62.5) |
| Educational attainment  (High school or less / College / Postgraduate), n (%) | 89 (18.5) / 210 (43.6) / 183 (38.0) | 13 (9.9) / 61 (46.6) / 57 (43.5) | 11 (11.5) / 45 (46.9) / 40 (41.7) |
| Ethno-racial background (Black / White / Other), n (%) | 16 (3.3) / 456 (94.6) / 10 (2.1) | 3 (2.3) / 120 (91.6) / 8 (6.1) | 3 (3.1) / 87 (90.6) / 6 (6.2) |
| CDR (0 / 0.5 / >0.5), n (%) | 0 (0.0) / 428 (88.8) / 54 (11.2) | 0 (0.0) / 123 (90.4) / 13 (9.6) | 0 (0.0) / 66 (68.8) / 30 (31.2) |
| CDR at last visit (0 / 0.5 / >0.5), n (%) | 0 (0.0) / 293 (60.8) / 189 (39.2) | 0 (0.0) / 118 (90.1) / 13 (9.9) | 0 (0.0) / 91 (94.8) / 5 (5.2) |
| CDR-SB | 2.17 ± 1.59 | 1.98 ± 1.54 | 1.79 ± 1.28 |
| CDR-SB at last visit | 4.18 ± 3.3 | 3.90 ± 3.6 | 3.6 ± 3.4 |
| mPACC | -8.70 ± 5.75 | -8.69 ± 5.82 | -8.11 ± 5.73 |
| mPACC at last visit | -13.1 ± 8.81 | -11.8 ± 8.28 | -11.2 ± 8.15 |
| Amyloid PET, Centiloid | 79.1 ± 31.0 | 70.5 ± 46.8 | 75.0 ± 31.2 |
| Amyloid PET-positive^a^, n (%) | 390 (94.9) | 96 (81.4) | 83 (93.3) |
| Mesial-temporal tau PET, SUVR | 1.71 ± 0.44 | 1.71 ± 0.26 | 1.73 ± 0.28 |
| Mesial-temporal tau PET-positive^b^, n (%) | 70 (73.7) | 82 (94.3) | 57 (93.4) |
| Estimated age at amyloid PET positivity, years | 61.2 ± 9.8 | 60.7 ± 9.6 | 60.7 ± 9.6 |
| Amyloid age group (< 65 / 65-75 / > 75), n (%) | 317 (65.8) / 118 (24.5) / 47 (9.8) | 67 (69.8) / 20 (20.8) / 9 (9.4) | 67 (69.8) / 20 (20.8) / 9 (9.4) |
| Estimated age at tau PET positivity, years | 68.3 ± 8.99 | 69.0 ± 9.24 | 68.3 ± 8.99 |
| Tau age group (< 65 / 65-75 / > 75), n (%) | 36 (35.6) / 37 (38.5) / 23 (24.0) | 45 (34.4) / 50 (38.2) / 36 (27.5) | 36 (37.5) / 37 (38.5) / 23 (24.0) |
| Amyloid-tau time interval | - | - | 7.6 ± 6.9 |
| Amyloid-tau time interval group (Tau first / Concurrent amyloid-tau / Amyloid first < 10 years / Amyloid first > 10 years) | - | - | 5 (5.3) / 22 (22.9) / 32 (33.3) / 37 (38.5) |
| Vascular risk factors^c^ | | | |
| Diabetes, n (%) | 33 (9.0) | 3 (6.4) | 3 (8.6) |
| Hypertension, n (%) | 199 (41.5) | 53 (40.8) | 38 (40.0) |
| Obesity, n (%) | 70 (14.6) | 23 (17.8) | 18 (18.9) |
| Dyslipidemia, n (%) | 131 (35.7) | 17 (36.2) | 11 (31.4) |
| Chronic kidney disease, n (%) | 94 (25.6) | 6 (12.8) | 4 (11.4) |
| Total white matter hyperintensity volume, % | 0.55 ± 0.72 | 0.50 ± 0.87 | 0.51 ± 0.94 |

Data are presented as mean ± standard deviation or n (%). Participants were classified as cognitively impaired if they had two consecutive visits with a CDR > 0 and a CDR > 0 at their last visit. Data from their first visit with a CDR > 0 is reported unless otherwise specified. 411 and 95 participants with estimated amyloid PET positivity age had an amyloid PET and a tau PET within a year of their first CDR 0 visit, respectively. 118 and 87 participants with estimated tau PET positivity age had an amyloid PET and a tau PET within a year of their first CDR 0 visit, respectively. 96 participants had estimates for both amyloid and tau PET positivity age and overlapped between the two cohorts. Of those, 89 and 61 had an amyloid PET and a tau PET within a year of their first CDR 0 visit, respectively. ^a^Amyloid PET positivity threshold: > 0.78 SUVR for FBP scans and > 0.74 SUVR for FBB scans; ^b^Mesial-temporal tau PET positivity threshold: > 1.41 SUVR.

^c^Obesity and hypertension data correspond to measurements obtained within one year of the amyloid PET acquisition. Participants were classified as obese when BMI > 30 kg/m², and as hypertensive when systolic blood pressure ≥ 130 mmHg and diastolic blood pressure ≥ 80 mmHg. Medical history of chronic kidney disease, diabetes, and dyslipidemia was collected at study entry and was available for 367 participants with estimated age at amyloid PET positivity, 47 with estimated age at tau PET positivity, and 35 with both estimates. White matter hyperintensity volume (cm³) was normalized to total intracranial volume and is expressed as a percentage.

# **Supplementary Table ST7. Rate of decline after symptom onset by biomarker positivity age group or amyloid-tau time interval group in ADNI**

|  | **Rate of change in CDR-SB (CDR-SB/year)** | **‘Time x group’ interaction** | | **‘Time x group x sex’ interaction** | | **‘Time x group x *APOE*-ε4’ interaction** | |
| --- | --- | --- | --- | --- | --- | --- | --- |
|  | **β [95% CI]** | **F (df1, df2)** | ***P*-value** | **F (df1, df2)** | ***P*-value** | **F (df1, df2)** | ***P*-value** |
| **Amyloid age group** | | 5.40  (2, 349) | 0.005* | 2.63  (2, 348) | 0.07 | 1.19  (2, 355) | 0.31 |
| < 65 | 0.96  [0.84 - 1.09] |  |  |  |  |  |  |
| 65 - 75 | 0.78  [0.57 - 0.98] |  |  |  |  |  |  |
| > 75 | 0.41  [0.08 - 0.73]^a^ |  |  |  |  |  |  |
| **Tau age group** | | 3.77  (2, 102) | 0.026* | 0.29  (2, 97) | 0.75 | 0.82  (2, 99) | 0.44 |
| < 65 | 1.07  [0.74 -1.39] |  |  |  |  |  |  |
| 65 - 75 | 0.72  [0.41 - 1.02] |  |  |  |  |  |  |
| > 75 | 0.39  [0.03 - 0.76]^a^ |  |  |  |  |  |  |
| **Amyloid-tau**  **time interval group** | | 0.98  (3, 76) | 0.41 | 0.71  (3, 70) | 0.55 | 0.98  (3, 73) | 0.41 |
| Concurrent amyloid-tau | 0.83  [0.41 - 1.26] |  |  |  |  |  |  |
| Amyloid first < 10 years | 0.81  [0.44 - 1.18] |  |  |  |  |  |  |
| Amyloid first > 10 years | 0.47  [0.14 - 0.81] |  |  |  |  |  |  |

Change in CDR-SB per year [95% CI] in participants after symptom onset (first visit with a CDR > 0). Participants were classified by age at biomarker positivity group or by amyloid-tau time interval group. Linear mixed effect (LME) models with random slopes and intercepts were used to assess the effect of biomarker positivity age or amyloid-tau time interval on the rates of cognitive decline over five years following symptom onset. Interaction terms between time since symptom onset and biomarker age group or interval group were evaluated. Next, pairwise contrasts were conducted to compare group-specific cognitive decline rates. Separate models included interactions between time, biomarker positivity age or amyloid-tau time interval group and sex or *APOE*-ε4 status. In the amyloid-tau time interval analyses, the tau-first group was excluded due to the small sample size (n=6). *Statistically significant (*P*<0.05). ^a^ *P*<0.05 *vs.* < 65 years

# **Supplementary Figure S1. Model fits to estimate amyloid and mesial-temporal tau PET clocks in ADNI**

Generalized Additive Models (GAM) with cubic spline were applied to evaluate the relationship between amyloid or mesial-temporal (entorhinal, parahippocampus, and amygdala) tau burden halfway the follow-up period (midpoint SUVR) and the rate of change in amyloid or mesial-temporal tau PET. For amyloid PET, ^18^F-florbetaben (n=204) and ^18^F-florbetapir (n=784) data were modelled separately. **A, D and G** show the GAM model fit including the entire midpoint SUVR range for amyloid (A and D) and tau PET (G) data. **B, E and H** show the variance of the GAM fitted values across midpoint SUVR values for amyloid (B and E) and tau PET (H) data. Horizontal dashed red line depicts the 90th percentile variance cutoff. **C, F and I** show the GAM model fit after excluding midpoint SUVR values above the 90th percentile variance cutoff, which resulted in an interval of 0.63-1.19 SUVR for ^18^F-florbetaben amyloid PET, 0.62-1.11 SUVR for ^18^F-florbetapir amyloid PET, and 0.98-2.04 SUVR for tau PET. Each point depicts the value of an individual and the solid blue lines indicate the model fit. *APOE*-ε4 carriers are shown in blue and *APOE*-ε4 non-carriers are shown in green.

# **Supplementary Figure S2. Validation of the amyloid and mesial-temporal tau PET clock estimates in ADNI**


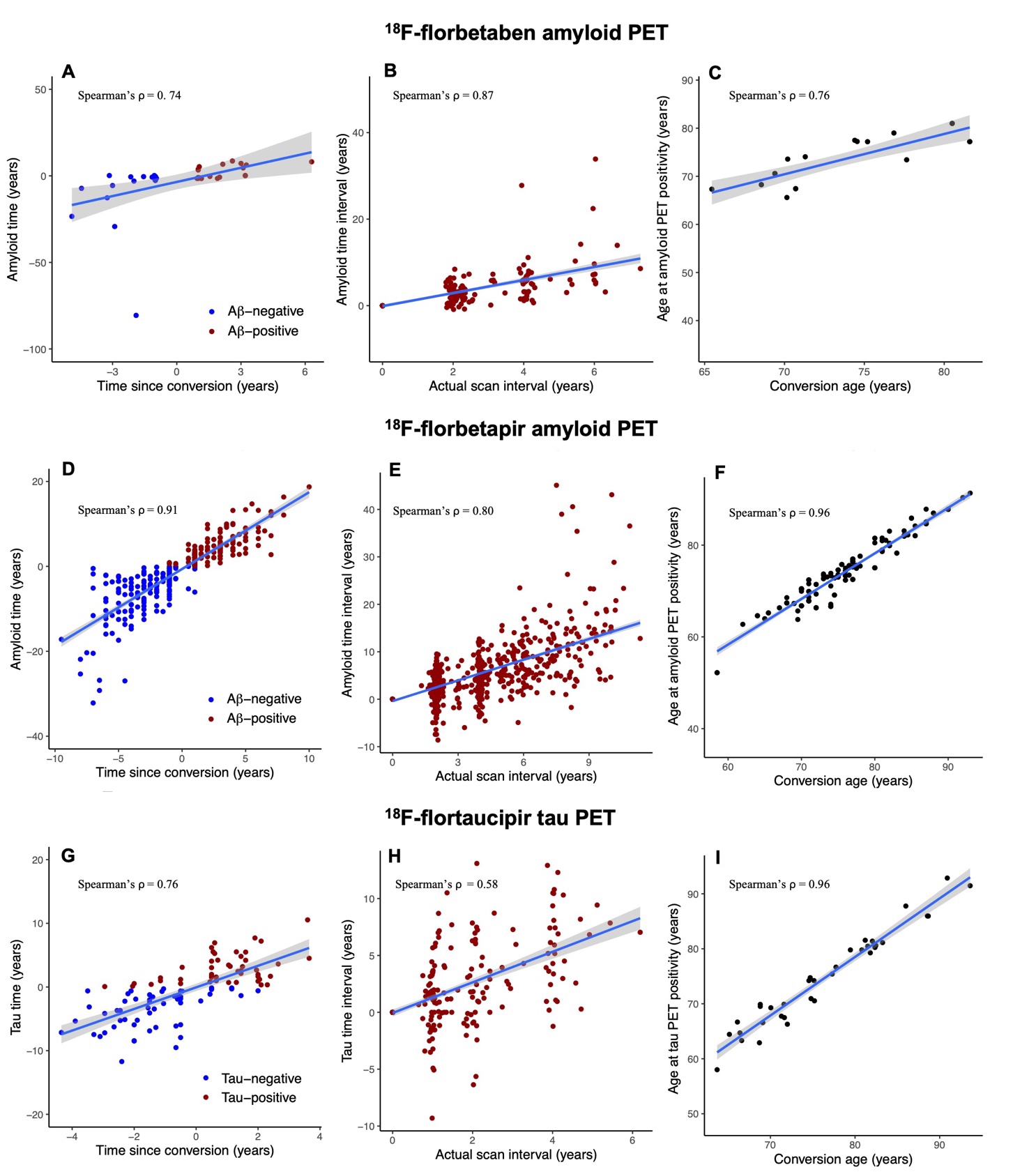


Amyloid and tau time models were validated in the group of individuals that converted from a negative to a positive amyloid or mesial-temporal tau PET scan throughout the study duration, respectively (PET converters; n=81 for FBP amyloid, n=14 for FBB amyloid, and n= 35 for FTP tau). Actual conversion age in PET converters was calculated by averaging the ages at the last negative scan and at the first positive scan. **A, D and G** show the correlation between the estimated amyloid time (A and D) or tau time (G) and the actual time since conversion. **B, E and H** show the correlation between amyloid (B and E) or tau (H) time intervals (amyloid or tau time - amyloid or tau time at first scan) and actual time intervals between scans above the positivity threshold in the whole sample. **C, F and I** show the correlations between the estimated age at amyloid PET positivity (C and F) or tau PET positivity (I) and the actual age at conversion in PET converters. Of note, each point at A, B, D, E, G and H depicts a scan and each point at C, F and I depicts an individual. Solid lines indicate the linear regression lines. Amyloid and tau PET positivity cutoffs were derived using Gaussian mixture modelling (GMM). For amyloid, a global cortical amyloid PET SUVR of 0.78 for FBP and of 0.74 SUVR for FBB were set as time 0. For tau, a mesial-temporal meta-ROI tau PET SUVR of 1.41 was set as time 0.

# **Supplementary Figure S3. Model fits and validation of the amyloid PET clock in the HABS**

**
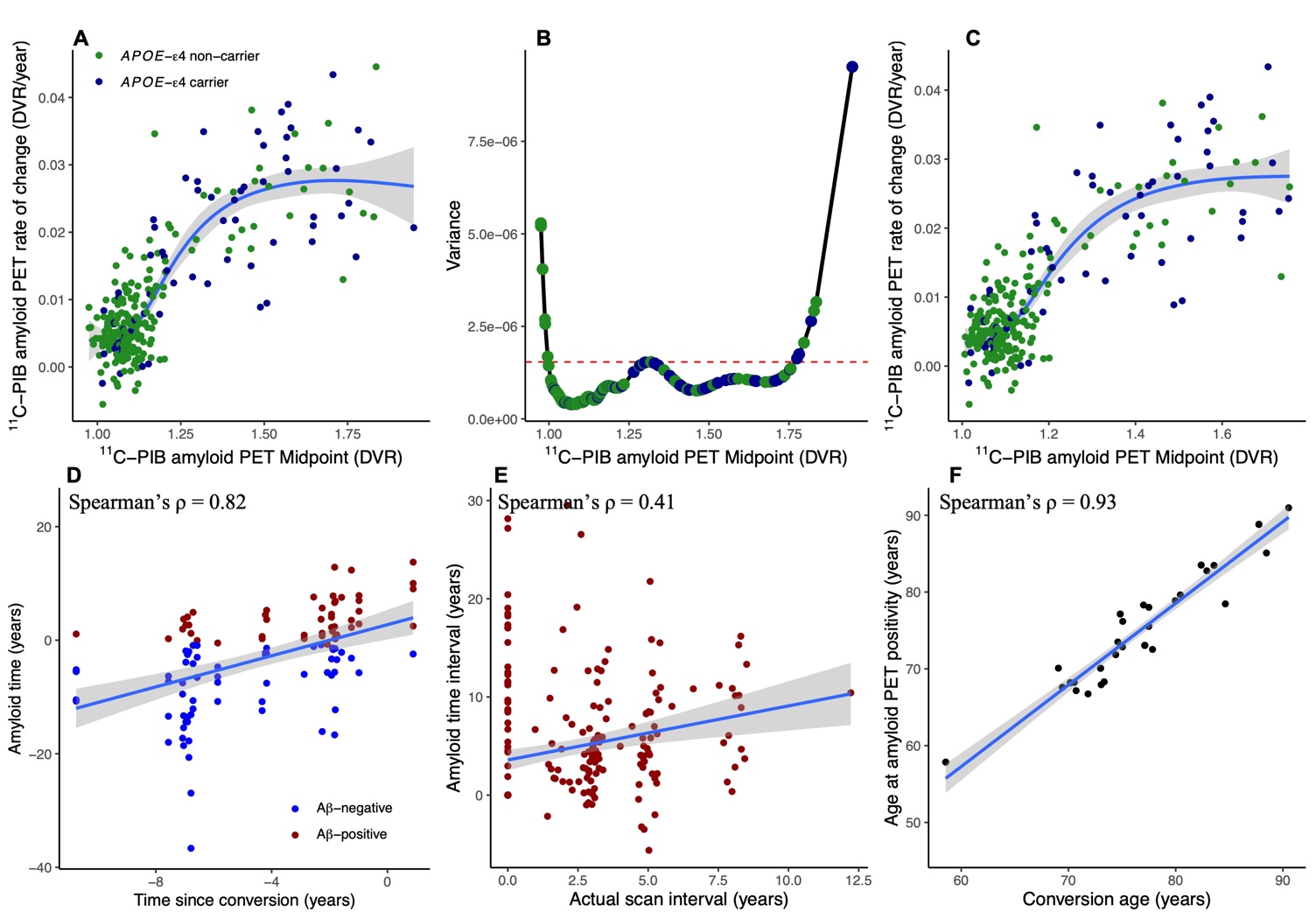
**

Generalized Additive Models (GAM) with cubic spline were applied to evaluate the relationship between amyloid PET burden halfway the follow-up period (midpoint DVR) and the rate of change in amyloid PET. **A** shows the GAM model fit including the entire midpoint DVR range. **B** shows the variance of the GAM fitted values across amyloid PET midpoint values. Horizontal dashed red line depicts the 90th percentile variance cutoff. **C** shows the GAM model fit after excluding amyloid PET midpoint DVR values above the 90th percentile variance cutoff, which resulted in an interval of 1.0-1.77 DVR. Amyloid PET time models were validated in the group of individuals that converted from a negative to a positive scan throughout the study duration (n=32). Actual conversion age in amyloid PET converters was calculated by averaging the ages at the last negative scan and at the first positive scan. **D** shows the correlation between the estimated amyloid PET time and the actual time since conversion for each scan. **E** shows the correlation between estimated amyloid PET time intervals between scans (amyloid time – amyloid time at first scan) and actual time intervals between scans above the positivity threshold in the whole sample. **F** shows the correlation between the estimated age at amyloid PET positivity and the actual age at conversion in amyloid PET converters. Of note, each point at D and E depicts a scan and each point at A, B, C and F depicts an individual. Solid lines indicate the linear regression lines. A global cortical (frontal, lateral temporal and retrospenial) amyloid PET DVR of 1.20 was set as time 0.

# **Supplementary Figure S4. Model fits and validation of the tau PET clock in the HABS**

**
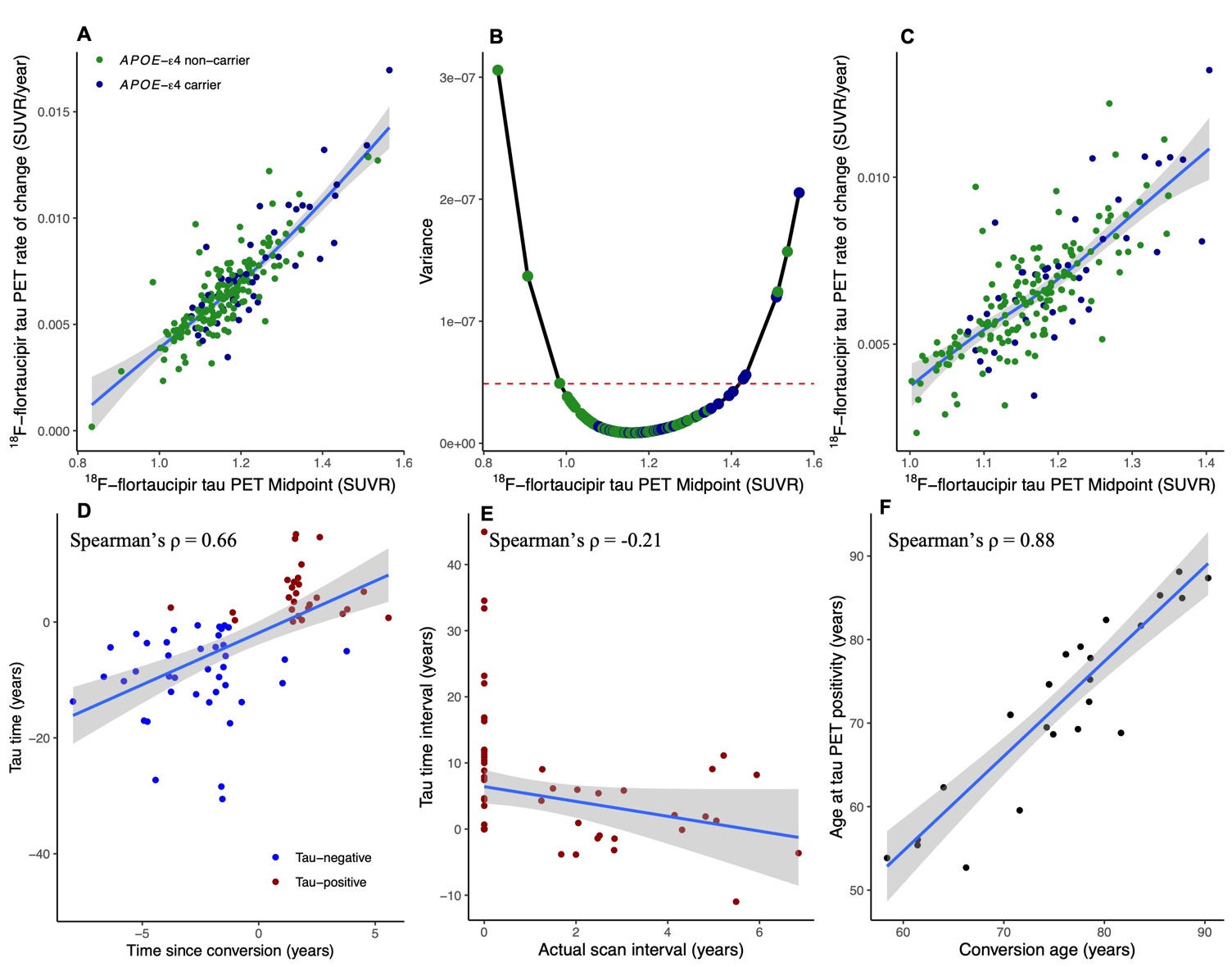
**

Generalized Additive Models (GAM) with cubic spline were applied to evaluate the relationship between tau PET burden halfway the follow-up period (midpoint SUVR) and the rate of change in tau PET. **A** shows the GAM model fit including the entire midpoint SUVR range. **B** shows the variance of the GAM fitted values across tau PET midpoint values. Horizontal dashed red line depicts the 90th percentile variance cutoff. **C** shows the GAM model fit after excluding tau PET midpoint SUVR values above the 90th percentile variance cutoff, which resulted in an interval of 0.99-1.42 SUVR. Tau PET time models were validated in the group of individuals that converted from a negative to a positive scan throughout the study duration (n=23). Actual conversion age in tau PET converters was calculated by averaging the ages at the last negative scan and at the first positive scan. **D** shows the correlation between the estimated tau PET time and the actual time since conversion for each scan. **E** shows the correlation between estimated tau PET time intervals between scans (tau time – tau time at first scan) and actual time intervals between scans above the positivity threshold in the whole sample. **F** shows the correlation between the estimated age at tau PET positivity and the actual age at conversion in tau PET converters. Of note, each point at D and E depicts a scan and each point at A, B, C and F depicts an individual. Solid lines indicate the linear regression lines. A Gaussian mixture model (GMM)-derived positivity cutoff of mesial-temporal meta-ROI tau PET SUVR > 1.27 was set as time 0.

# **Supplementary Figure S5. Model fits and validation of the ADNI plasma p-tau217 clock**

**
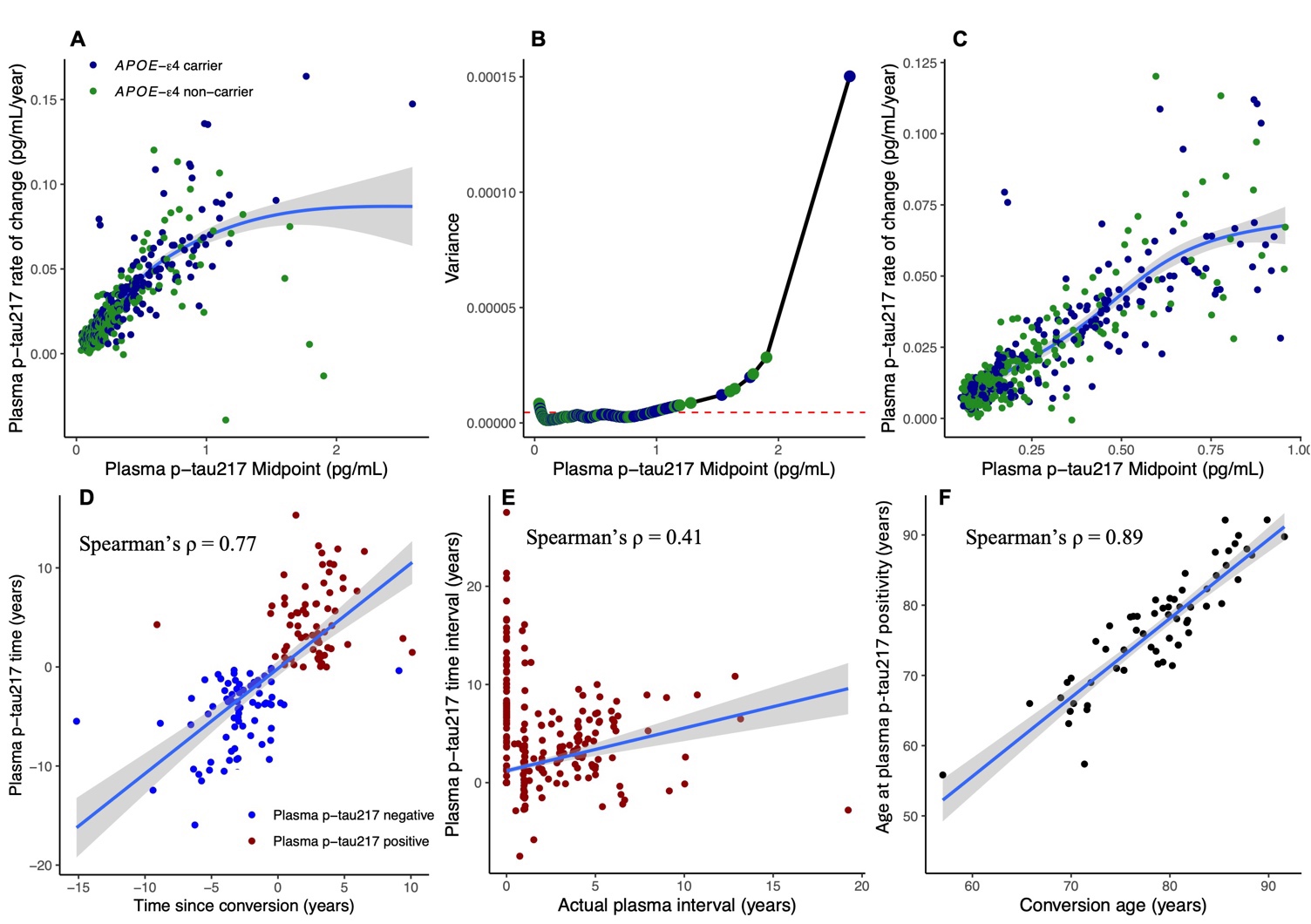
**

Generalized Additive Models (GAM) with cubic spline were applied to evaluate the relationship between plasma p-tau217 burden halfway the follow-up period (plasma p-tau217 midpoint) and the rate of change in plasma p-tau217. **A** shows the GAM model fit including the entire midpoint values range. **B** shows the variance of the GAM fitted values across midpoint plasma p-tau217 values. Horizontal dashed red line depicts the 90th percentile variance cutoff. **C** shows the GAM model fit after excluding plasma p-tau217 midpoint values above the 90th percentile variance cutoff, which resulted in an interval of 0.052-0.96 pg/mL. Plasma p-tau217 time models were validated in the group of individuals that converted from a negative to a positive plasma p-tau217 value throughout the study duration (n=67). Actual conversion age in plasma converters was calculated by averaging the ages at the last negative plasma measure and at the first positive plasma measure. **D** shows the correlation between the estimated plasma p-tau217 time and the actual time since conversion for each plasma measure. **E** shows the correlation between estimated plasma p-tau217 time intervals (plasma p-tau217 time – plasma p-tau217 time at first scan) and actual time intervals between plasma measures above the positivity threshold in the whole sample. **F** shows the correlation between the estimated age at plasma p-tau217 positivity and the actual age at conversion in plasma converters. Of note, each point at D and E depicts a scan and each point at A, B, C and F depicts an individual. Solid lines indicate the linear regression lines. A Gaussian mixture model (GMM)-derived positivity cutoff of 0.257 pg/mL was set as time 0 for the plasma p-tau217 clock.

# **Supplementary Figure S6. Estimated ages at amyloid and tau PET positivity by *APOE*-ε4 status and sex in the HABS**

Estimated ages at amyloid (**A**) or tau (**B**) PET positivity by *APOE*-ε4 status and sex. Box plots depict median (horizontal bar), IQR (hinges), and 1.5 × IQR (whiskers). Beta coefficients and *P*-values from linear regression models are reported for the main effects of *APOE*-ε4 status (carriers *vs.* non-carriers) or sex (women *vs.* men). Interaction terms were not evaluated in HABS due to the limited statistical power.

# **Supplementary Figure S7. Characteristics of the amyloid-tau time interval in the HABS**

**
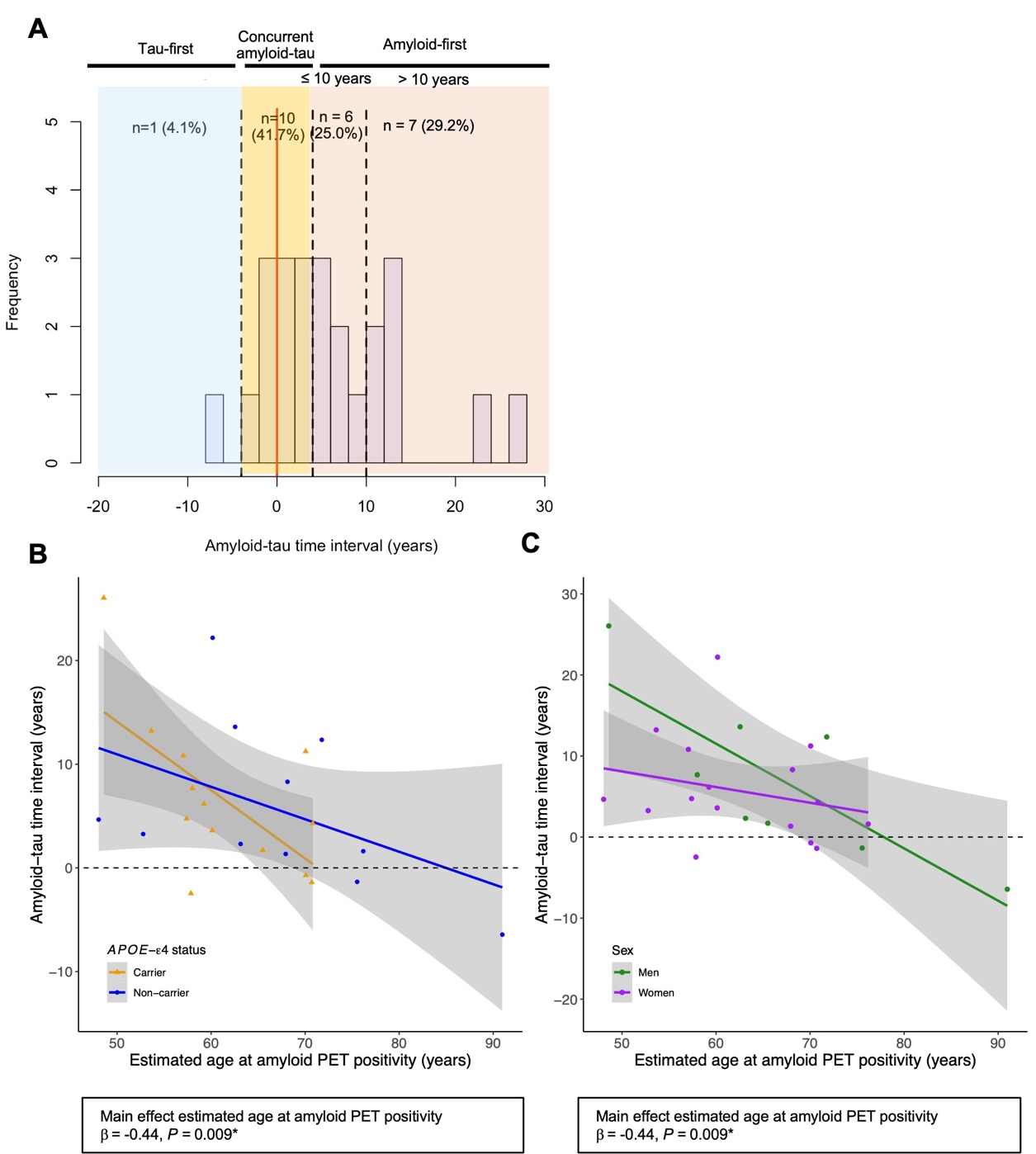
**

**A.** Distribution of the amyloid-tau time interval. The “concurrent amyloid-tau” group was defined by an interval of ± 4 years, reflecting cumulative mean absolute error for estimated positivity ages (see Supplementary Methods). Participants that became amyloid-positive first and tau-positive 4 to 10 years later were classified as “Amyloid first ≤ 10 years”, and participants that became amyloid-positive first and tau-positive more than 10 years later were classified as “Amyloid-first > 10 years”. **B, C**. Scatter plots show the association between estimated age at amyloid PET positivity and the amyloid-tau time interval by *APOE*-ε4 status (B) and sex (C). Solid lines indicate the regression line and 95% CIs. The dashed horizontal line at 0 years indicates concurrent amyloid and tau PET positivity. Positive values reflect individuals who became tau PET positive after amyloid PET positivity; negative values indicate tau PET positivity preceding amyloid PET positivity. Beta coefficients and *P*-values from linear regression models are reported for the main effects of estimated age at amyloid PET positivity. All models included *APOE*-ε4 status and sex as covariates. Main effects of sex or *APOE*-ε4 status and interaction were not evaluated in HABS due to the limited statistical power.

# **Supplementary Figure S8. Amyloid-plasma p-tau217 time interval distribution in ADNI**

**
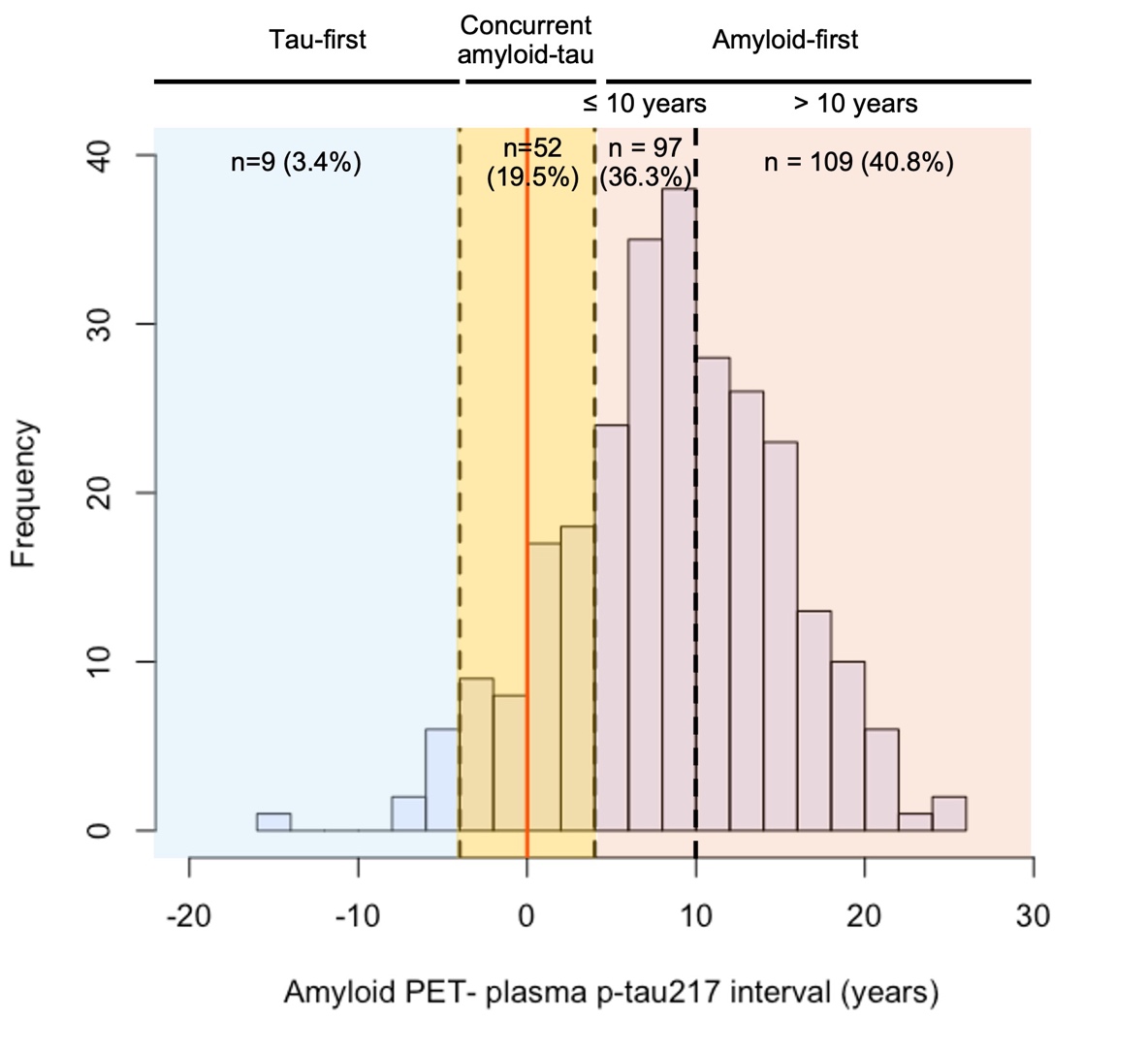
**

Distribution of the amyloid-plasma p-tau217 time interval. The “concurrent amyloid-tau” group was defined by an interval of ±4 years, reflecting cumulative mean absolute error for estimated positivity ages (see Supplementary Methods). Participants that became amyloid-positive first and plasma p-tau217-positive 4 to 10 years later were classified as “Amyloid first ≤ 10 years”, and participants that became amyloid-positive first and plasma p-tau217-positive more than 10 years later were classified as “Amyloid-first > 10 years”.

# **Supplementary Figure S9. Effect of estimated biomarker positivity age and amyloid-tau time interval on time to symptom onset following amyloid or tau PET positivity in ADNI**

Forest plots displaying time ratios and 95% confidence intervals (CI) from Accelerated Failure Time (AFT) models examining time from amyloid or tau PET positivity to CDR > 0. A time ratio > 1 indicates slower progression (longer time to impairment), whereas a time ratio < 1 indicates faster progression (shorter time to impairment). **A** and **B** include the effects of the estimated age at amyloid PET positivity or tau PET positivity on progression rate in the amyloid or tau timeline, respectively. **C** and **D** include the effects of the amyloid-tau time interval on progression rate in the amyloid or tau timeline, respectively. All models included sex, *APOE*-ε4 status and educational attainment as covariates.

# **Supplementary Figure S10. Effect of estimated biomarker positivity age and amyloid-tau time interval on time to symptom onset following amyloid or tau PET positivity in the HABS**

Forest plots displaying time ratios and 95% confidence intervals (CI) from Accelerated Failure Time (AFT) models examining time from amyloid or tau PET positivity to CDR > 0. A time ratio > 1 indicates slower progression (longer time to impairment), whereas a time ratio < 1 indicates faster progression (shorter time to impairment). **A** and **B** include the effects of the estimated age at amyloid PET positivity or tau PET positivity on progression rate in the amyloid or tau timeline, respectively. **C** and **D** include the effects of the amyloid-tau time interval on progression rate in the amyloid or tau timeline, respectively. All models included sex, *APOE*-ε4 status and educational attainment as covariates.

# **Supplementary Figure S11. Clinical progression after symptom onset by baseline chronological age group in ADNI**

Linear mixed-effects models with random intercepts and slopes were used to model CDR-SB trajectories as a function of years since symptom onset (defined as the first visit with CDR > 0), grouping individuals by baseline chronological age group**.** The interaction term between time and baseline age group was evaluated to assess differences in the rate of cognitive decline. Individual trajectories are shown, where each line connects multiple observations from the same individual.

# **Supplementary Figure S12. Model fits and validation of the ADNI temporo-parietal tau PET clock**


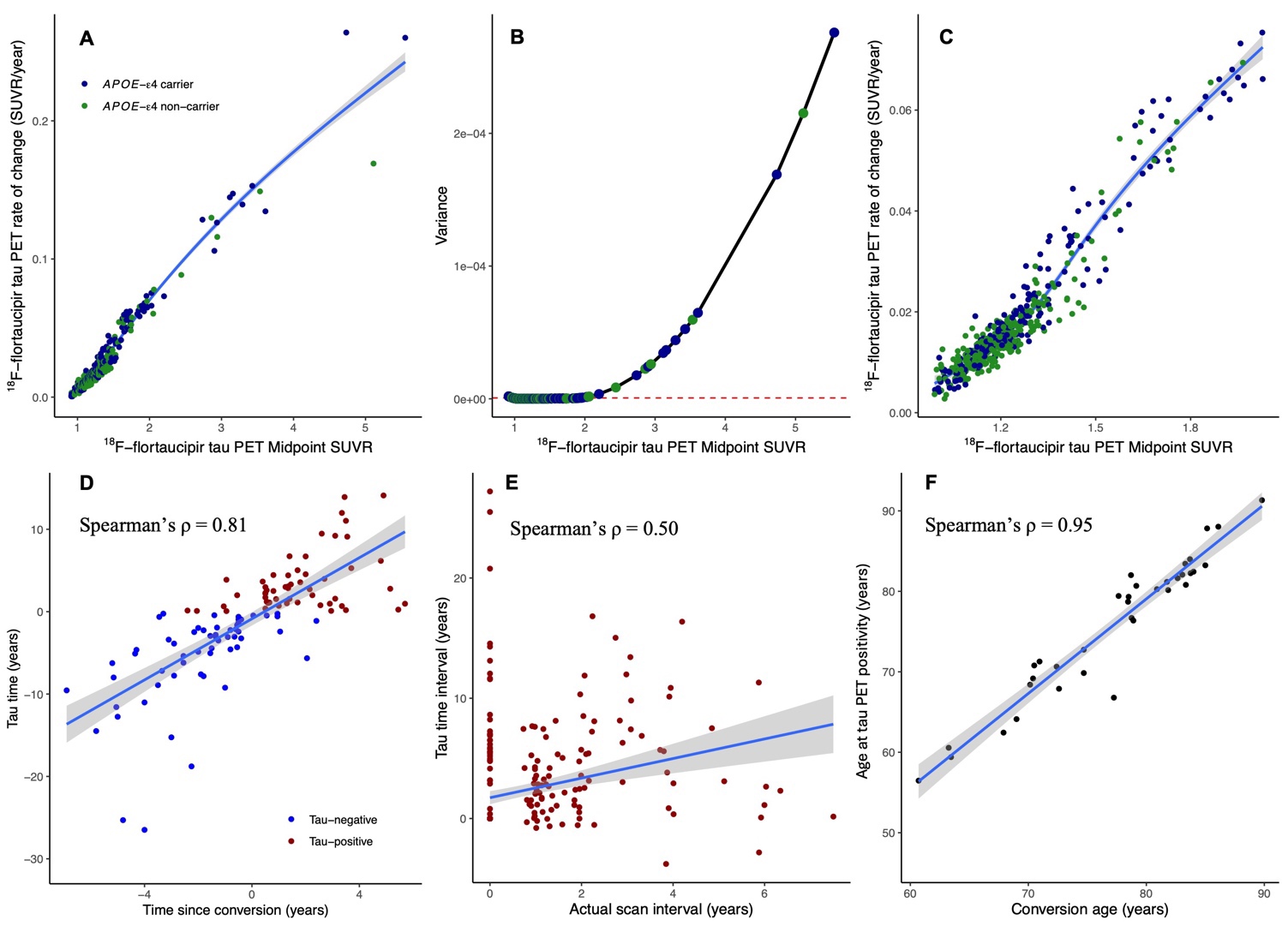


Generalized Additive Models (GAM) with cubic spline were applied to evaluate the relationship between temporo-parietal tau PET burden halfway the follow-up period (tau PET midpoint SUVR) and the rate of change in tau PET. **A** shows the GAM model fit including the entire midpoint SUVR range. **B** shows the variance of the GAM fitted values across tau PET midpoint values. Horizontal dashed red line depicts the 90th percentile variance cutoff. **C** shows the GAM model fit after excluding tau PET midpoint SUVR values above the 90th percentile variance cutoff, which resulted in an interval of 0.99-2.03 SUVR. Temporo-parietal tau PET time models were validated in the group of individuals that converted from a negative to a positive scan throughout the study duration (n=35). Actual conversion age in tau PET converters was calculated by averaging the ages at the last negative scan and at the first positive scan. **D** shows the correlation between the estimated tau PET time and the actual time since conversion for each scan. **E** shows the correlation between estimated tau PET time intervals between scans (tau time – tau time at first scan) and actual time intervals between scans above the positivity threshold in the whole sample. **F** shows the correlation between the estimated age at tau PET positivity and the actual age at conversion in tau PET converters. Of note, each point at D and E depicts a scan and each point at A, B, C and F depicts an individual. Solid lines indicate the linear regression lines. The temporo-parietal meta-ROI included the following regions: banks of superior temporal, cuneus, inferior–superior parietal, inferior-middle-superior temporal, istmuscingulate, lateral occipital, lingal, posterior cingulate, precuneus, and supramarginal. A Gaussian mixture model (GMM)-derived positivity cutoff of 1.35 SUVR was set as time 0 for the temporo-parietal tau PET clock.

# **Supplementary Figure S13. Effects of *APOE*-ε4 status and sex across disease progression milestones in ADNI**

Forest plots summarizing the associations of sex (women *vs.* men) and *APOE*-ε4 status (carriers *vs.* non-carriers), and their interaction, on estimated ages at amyloid (A) and tau (B) PET positivity, the amyloid-tau time interval (C), and on time ratios from AFT models assessing time to symptom onset following amyloid (D) and tau (D) PET positivity. Beta coefficients (A–C) and time ratios (D–E) with corresponding 95% confidence intervals are displayed. Models in C, D and E were further adjusted for biomarker positivity age and educational attainment. **P*-value<0.05
